# Extraction and Identification of the Bioactive Metabolites Produced by *Curvularia inaequalis*, an Endophytic Fungus Collected in Iran from *Echium khuzistanicum* Mozaff

**DOI:** 10.3390/molecules30193870

**Published:** 2025-09-24

**Authors:** Maryam Besharati, Maria Letizia Ciavatta, Marianna Carbone, Nadia Cacciapuoti, Martina Aversa, Emanuela Roscetto, Stefany Castaldi, Giancarlo Perrone, Angela Boari, Katia Gialluisi, Maria Rosaria Catania, Sayed Ali Moosawi-Jorf, Antonio Evidente

**Affiliations:** 1 Institute of Biomolecular Chemistry (ICB), National Research Council (CNR), 80078 Pozzuoli, Italy; besharatimaryam513@yahoo.com (M.B.); marianna.carbone@cnr.it (M.C.); nadia.cacciapuoti@cnr.it (N.C.); evidente@unina.it (A.E.); 2 Faculty of Agriculture, Tarbiat Modares University, Tehran 14115-111, Iran; moosawijorf@modares.ac.ir; 3 Department of Molecular Medicine and Medical Biotechnologies, University of Naples Federico II, 80131 Napoli, Italy; martina.aversa@unina.it (M.A.); emanuela.roscetto@unina.it (E.R.); mariarosaria.catania@unina.it (M.R.C.); 4 Department of Biology, University of Naples Federico II, 80126 Napoli, Italy; stefany.castaldi@unina.it; 5 Institute of Sciences of Food Production (ISPA), National Research Council (CNR), 70125 Bari, Italy; giancarlo.perrone@cnr.it (G.P.); angela.boari@cnr.it (A.B.); katia.gialluisi@cnr.it (K.G.)

**Keywords:** *Echium khuzistanicum*, endophyte fungi, *Curvularia inaequalis*, secondary metabolites, biological activity

## Abstract

Endophytic fungi (EF) are microorganisms that colonize the internal tissues of host plants, providing a range of benefits to them. In this symbiosis, they act as a reservoir of bioactive metabolites that are important for enhancing the host’s defense mechanisms as a resistance against pathogens. These molecules usually possess antimicrobial properties that can be exploited for application in agriculture and medicine. In this context, the current work was designed to evaluate the phytotoxic and antimicrobial properties of the endophytic fungus *Curvularia inaequalis*, isolated for the first time from the Iranian medicinal plant *Echium khuzistanicum*. Culture filtrates, their organic extracts, and isolated metabolites were tested against a series of plants to assess their phytotoxicity, as well as against a wide range of plant and human pathogens to evaluate their antimicrobial activity. The main compounds characterizing the organic extract of *C. inaequalis* have been identified as (*R*)-phomalactone, catenioblin A, and (-) asperpentyn (**1**–**3**) by using spectroscopic techniques, NMR mainly, and HR-ESI-MS. In the bioactivity evaluation carried out in this study, (*R*)-phomalactone (**1**) stood out as the most promising compound, exhibiting significant non-host phytotoxic activity on tomato leaves; potent antibacterial activity against a wide range of human pathogens, including methicillin-resistant *Staphylococcus aureus* (MRSA) strains; and marked antifungal activity against several economically important phytopathogens. (–)-Asperpentyn (**3**) also showed robust and selective antifungal activity against phytopathogens, while catenioblin A (**2**) exhibited only a slight phytotoxic effect and limited overall bioactivity in this study. These findings reveal that the isolated endophytic fungi hold considerable promise as an untapped source of bioactive metabolites with antibacterial, antifungal, and phytotoxic activities.

## 1. Introduction

Microorganisms have long been recognized as a huge reserve of biodiversity and a nearly endless supply of structurally varied bioactive secondary metabolites, with many proving useful in different fields [1,2,3,4,5,6,7,8]. Among these, since the early 90s, endophytes have attracted remarkable interest as a new and alternative source of pharmaceutical and agrochemical products. They include fungi, bacteria, and actinomycetes that spend part or all of their life cycle within the tissue of plants without causing visible symptoms of diseases [8]. Endophytic fungi have been found in plants growing in diverse climatic regions worldwide and are commonly isolated from all plant organs [9]. This group of microorganisms, known since 1904, garnered significant interest when paclitaxel was isolated from *Taxomyces andreanae*, an endophytic fungus found in the inner bark of *Taxus brevifolia*. More than 200 endophytic fungi belonging to several taxa have been grown for the production of paclitaxel and/or its derivatives [10,11,12,13,14]. Metabolites produced by endophytic fungi have been recently reviewed [15].

Given the pressing need for searching novel sources of bioactive compounds, and based on the substantial premises reported above, our study focused on the previously unexplored fungal community of *Echium khuzistanicum* Mozaff. This plant, also known as “viper’s bugloss”, holds significant cultural and medicinal value. It is widely used in Iranian traditional medicine to treat a range of ailments, from respiratory diseases and ulcers to mental health issues and promoting wound healing. Beyond these established uses, research has also demonstrated the antioxidant, antibacterial, and anti-inflammatory properties of *E. khuzistanicum* extracts. Related plants belonging to the same genus, Echium, have been recognized for hosting diverse endophytic fungal species [16]. During our study, the fungus *Curvularia inaequalis* has been isolated from the leaves of *E. khuzistanicum* and characterized by using morphological and genetic techniques. The chemical investigation carried out into the extracts from this *C. inaequalis* strain, cultivated under different liquid conditions, led to the isolation of three main bioactive metabolites (**1**–**3**). These compounds have been identified by using spectroscopic and physical methods and their antimicrobial efficacy against multidrug-resistant human bacteria and plant pathogens as well as their phytotoxic effects have been evaluated.

## 2. Results and Discussion

**Figure 1:**
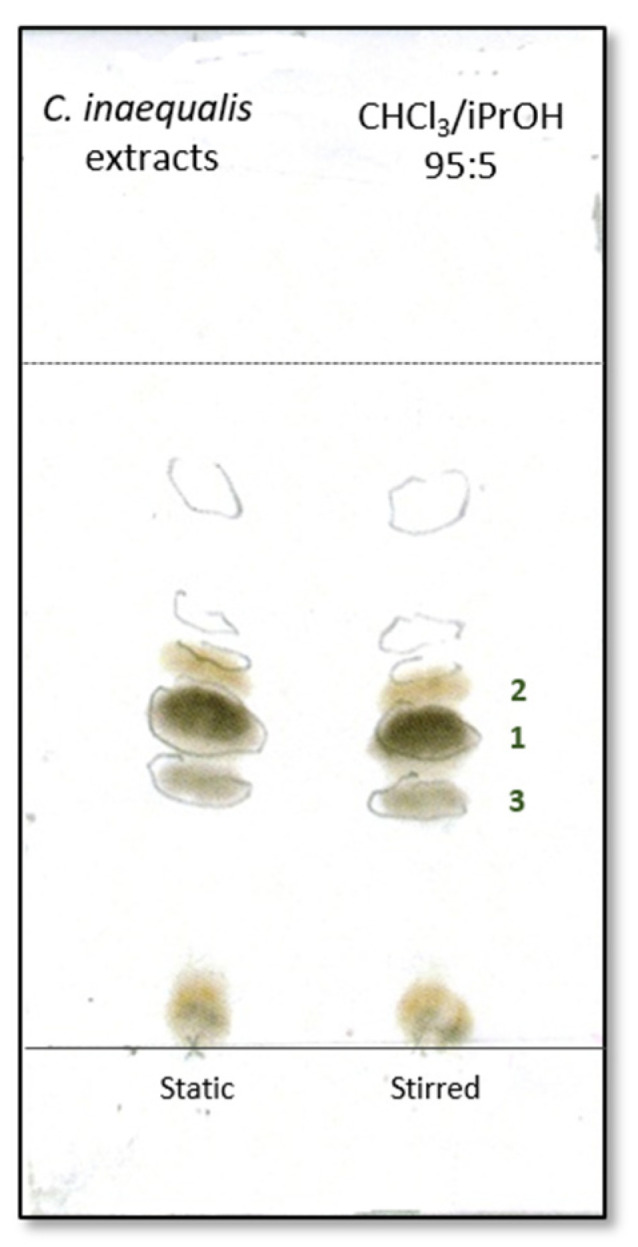
Metabolite profile of static and stirred culture filtrate extracts from *C. inaequalis*.

**Figure 2:**
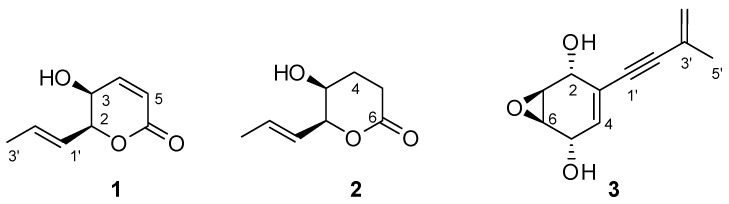
Structures of phomalactone, catenioblin A, and asperpentyn (**1**–**3**).

The *Curvularia inaequalis* strain was isolated in Iran from the plant *Echium khuzistanicum*. The fungus strain was transferred to Italy at ISPA (Institute of Sciences of Food Production) where it was identified using morphological and genetic techniques, and grown in both stirred and static liquid conditions, as different growth conditions were occasionally found to affect the composition and yield of the metabolites (see the Section 3). Both culture filtrates were preliminary tested by leaf puncture assay on mauve (*Calluna vulgaris* L.), vitriol grass (*Parietaria officinalis* L.), marigold (*Calendula officinale* L.), perennial sowthistle (*Sonchus arvensis* L.), and hemp (*Cannabis sativa* L.), exhibiting phytotoxic activity (see Materials and Methods) [17]. Successively, culture filtrates and micelia were transferred to ICB (Institute of Biomolecular Chemistry) where they were submitted to ethyl acetate extraction (EtOAc). To obtain an exhaustive extraction, cultures filtrates were first extracted at the original pH and subsequently in acid conditions. The organic extract of mycelia was not further worked. The organic extracts from culture filtrates were preliminary analyzed by TLC that revealed the presence of several UV-absorbing spots (Figure 1). As the metabolite profiles were found to be the same, these extracts were combined and purified as detailed reported in the Section 3. Three compounds—(*R*)-phomalactone (**1**), its 2,3-dihydroderivative catenioblin A (**2**), and the epoxyquininoid (–)-asperpentyn (**3**)—were isolated from the EtOAc extract. (*R*)-phomalactone (**1**) was the most abundant component of the extract, accounting for approximately 17% of its total composition. The structures of all isolated compounds (Figure 2) were elucidated using spectroscopic methods, mainly NMR, optical rotation, and mass spectrometry, and confirmed by comparison with published data [18,19,20,21].

Phomalactone (**1**): colorless oil; [α]_D_ −170.6 (c 0.5, CHCl_3_), lit −175.3 (c 0.42, CHCl_3_) ref. [19]; ^1^H-NMR (CDCl_3_): δ 6.98 (dd, *J* = 9.9, 5.3 Hz, 1H, H-4), 6.12 (d, *J* = 9.9 Hz, 1H, H-5), 6.01 (dq, *J* = 15.5, 6.9 Hz, 1H, H-2′), 5.73 (ddd, *J* = 15.5, 6.9, 2.6 Hz, 1H, H-1′), 4.83 (dd, *J* = 6.9, 2.6, 1H, H-2), 4.20 (br s, 1H, H-3), 1.81, (d, *J* = 6.9, 3H, H-3′); ^13^C NMR (CDCl_3_): 163.2 (C, C-6), 144.5 (CH, C-4), 133.1 (CH, C-2′), 123.8 (CH, C-1′), 122.8, (CH, C-5), 81.1, (CH, C-2), 63.1 (CH, C-3), 18.0 (CH_3_, C-3′); HR-(+)-ESIMS (positive ion mode) *m*/*z* 177.0519 [M+ Na] (calcd. 177.0528 for C_8_H_10_O_3_Na);

2,3-Dihydrophomalactone (**2**): colorless oil; [α]_D_ + 38.6 (c 0.1, CHCl_3_), lit +36.4 (c 0.14, CHCl_3_) ref. [20]; ^1^H-NMR (CDCl_3_): δ 5.85 (dq, *J* = 15.6, 6.0 Hz, 1H, H-2′), 5.51 (dd, *J* = 15.6, 6.9 Hz, 1H, H-1′), 4.43 (dd, *J* = 6.9, 6.0 Hz, 1H, H-2), 4.07 (app t, *J* = 6.6, 6.6 Hz, 1H, H-3), 2.57 (m, 1H, H-5a), 2.52 (m, 1H, H-5b), 2.22 (m, 1H, H-4a), 2.07 (m, 1H, H-4b), 1.73, (d, *J* = 6.0, 3H, H-3′); ^13^C NMR (CDCl_3_): 177.1 (C, C-6), 130.1 (CH, C-2′), 127.9 (CH, C-1′), 82.7, (CH, C-2), 74.9 (CH, C-3), 28.4 (CH_2_, C-5), 23.7 (CH_2_, C-4), 17.8 (CH_3_, C-3′); [α]_D_. HR-(+)-ESIMS (positive ion mode) *m*/*z* 179.0683 [M+ Na] (calcd. 177.0684 for C_8_H_12_O_3_Na).

(–)-Asperpentyn (**3**): colorless oil; [α]_D_ −32.8 (c 0.1, CHCl_3_), lit −35.0 (c 0.11, CHCl_3_) ref. [21]; ^1^H-NMR (CDCl_3_): δ 6.07 (d, *J* = 4.9 Hz, 1H, H-4), 5.35 (s, 1H, H-4a’), 5.30 (s, 1H, H-4b’), 4.52 (br d, *J* = 4.9 Hz, 1H, H-5), 4.50 (s, 1H, H-2), 3.40 (s, 1H, H-1), 3.33 (s, 1H, H-6) 1.91, (s, 3H, H-5′); ^13^C NMR (CDCl_3_): 131.3 (CH, C-4), 126.2 (C, C-3′), 123.2 (CH_2_, C-4′), 92.1 (C, C-2′), 86.9, (C, C-1′), 65.6 (CH, C-2), 62.8 (CH, C-5), 52.3 (CH, C-1), 51.4 (CH, C-6), 23.2 (CH_3_, C-5′); HR-(-)-ESIMS (negative ion mode) *m*/*z* 191.0706 [M-H] (calcd. 191.0714 for C_11_H_11_O_3_).

Apart from (*R*)-phomalactone (**1**) and (–)-asperpentyn (**3**), which were already isolated from a marine strain of *C. inaequalis* [21,22], catenioblin A (**2**) is here reported for the first time from this species. However, compound **2** and phomalactone have been previously isolated from a number of different fungal genera, among them the entomopathogenic fungi *Hirsutella thompsonii* var. *synnematosa* and *Nigrospora sacchari* [19,23] and *Xylaria* sp. and *Paecilomyces cateniobliquus* [24,25].

The three compounds are known to possess a wide range of biological properties, with (*R*)-phomalactone (**1**) having the most promising bioactivity profile. In particular, (*R*)-phomalactone (**1**) and catenioblin A (**2**) have been reported to have an inhibitory effect on the growth of *Helicoverpa armigera*, commonly known as the cotton bollworm, which is a major pest impacting numerous crops throughout Europe, Africa, Asia, Australia, and China [25]. In addition, compound **1** was also found to induce the mortality of the nematode *Meloidogyne incognita*, reaching 84% in 96 h at the concentration of 500 mg/L [19]. The phytotoxic activity of (*R*)-phomalactone is also well documented. In greenhouse experiments, this metabolite demonstrated potent herbicidal activity by inhibiting seedling growth and by causing electrolyte leakage from photosynthetic tissues of both *Zinnia elegans* leaves and cucumber cotyledons [26]. Furthermore, (*R*)-phomalactone is also known for its antifungal activity against several species that are pathogens of agrarian crops [27,28]. Both (*R*)-phomalactone and catenioblin A were also assessed for their anti-plasmodial activity against a chloroquine-resistant strain of *Plasmodium falciparum*, resulting in only compound **1** being active and thus suggesting that the unsaturated moiety present in (*R*)-phomalactone **1** is crucial for the activity [29]. Instead, asperpentyn (**3**) has been identified in the extract of a *Curvularia* sp. G6-32 culture filtrate that was found to have relevant antioxidant and anticholinesterasic activity and low antimicrobial action [30].

To expand upon their known biological activities, the three isolated compounds were here tested across a spectrum of assays to further investigate their potential in both agriculture and medicine. In particular, the phytotoxic activity of (*R*)-phomalactone, catenioblin A, and (–)-asperpentyn, (**1**–**3**), along with the crude extract of the *C. inaequalis* culture filtrate, was tested on a tomato (*Lycopersicon esculentum* L.) cutting, as reported in the Section 3. As shown in Figure S1, only the organic extract from the culture filtrate and phomalactone induced toxicity effects comparable to those of ophiobolin A, which was used as positive standard [31]. The phytotoxicity increased from the time of inoculation up to a marked wilting and necrosis after 72 h. These results are in accordance with the literature confirming that the unsaturated lactone is an important structural feature for this activity.

Pure compounds **1**–**3** as well as the crude extracts from *C. inequalis* were evaluated for their antifungal activity against a panel of virulent phytopathogenic fungi, including *Macrophomina phaseolina*, *Botrytis cinerea*, *Alternaria alternata*, and *Septoria nodorum*, that are responsible for severe diseases in economically important crops.

**Figure 3:**
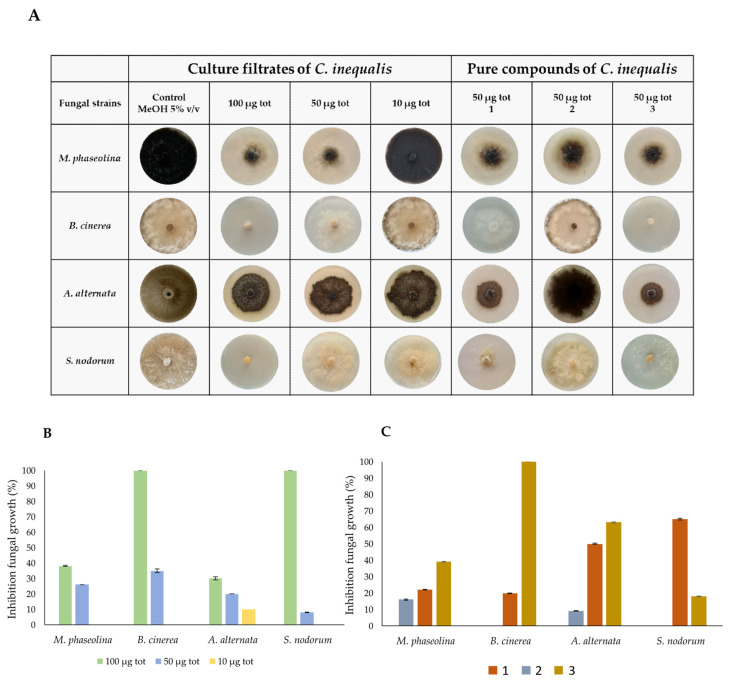
Antifungal activity. (**A**) Representative photographs for in vitro inhibition of mycelial growth of *M. phaseolina*, *B. cinerea*, *A. alternata*, and *S. nodorum* by culture filtrates (100, 50, and 10 μg tot) and three pure metabolites (**1**–**3**) purified from *C. inequalis*. (**B**) Percentage of inhibition of fungal growth by culture filtrates and (**C**) three pure metabolites (**1**–**3**). Graphs report the percentage reduction in the diameter of the fungal mycelia on the treated plate compared to the control plate, where fungi were grown alone. All experiments were performed in triplicate with three independent trials. Data are presented as means ± standard deviation.

The crude extract was tested at three different concentrations (5 μg/μL, 2.5 μg/μL, and 0.5 μg/μL). A volume of 20 μL of each sample, corresponding to total amounts of 100, 50, and 10 μg, respectively, was applied onto the fungal plugs. The inhibitory effect was evaluated in comparison with the control fungal plug treated with 20 μL of 5% *v*/*v* MeOH. As shown in Figure 3B, 100 μg of culture filtrate crude extract completely inhibited the growth of *B. cinerea* and *S. nodorum*, whereas the growth of *M. phaseolina* and *A. alternata* was reduced by approximately 40%. At a concentration of 50 μg, the growth of all fungal species decreased by about 30%, whereas no inhibitory effects were observed at the lowest tested concentration (10 μg). Pure (*R*)-phomalactone, catenioblin A, and (–)-asperpentyn (**1**–**3**) were assessed by using the minimal inhibitory concentrations (50 μg) determined for the crude extract. As shown in Figure 3A–C, (*R*)-phomalactone (**1**) and (–)-asperpentyn (**3**) exhibited strong inhibitory activity against all fungal species, while catenioblin A (**2**) was found to not be active. Notably, (*R*)-phomalactone (**1**) and (–)-asperpentyn (**3**) showed different selectivity, with compound **3** being more effective than **1** in inhibiting the growth of *M. phaseolina*, *B. cinerea*, and *A. alternata*, whereas (*R*)-phomalactone (**1**) displayed greater activity against *S. nodorum*.

To investigate their potential application in the biomedical field, the antimicrobial properties of compounds **1**–**3** and the crude extract of *C. inaequalis* culture filtrates were also assayed against several bacterial and fungal human pathogens, as detailed in the Section 3. The results reported in Table 1 indicate that the organic extract was active against all tested microbial strains, with growth inhibition values ≥ 90%. In particular, minimum concentrations inhibiting 100% growth (MIC_100_) for *Staphylococcus aureus* (ATCC 6538) and for *Candida albicans* (ATCC 10231) were determined at 250 µg/mL and 1 mg/mL, respectively, whereas for *Enterococcus faecalis* (ATCC 29212), *Acinetobacter baumannii* (ATCC BAA747), and *Pseudomonas aeruginosa* (ATCC 27853), MIC_90_ values were calculated at 1 mg/mL. Among the pure compounds, only (*R*)-phomalactone (**1**) exhibited activity. In particular, compound **1** showed a MIC_100_ of 62.5 µg/mL on the two strains of *S. aureus*, with bactericidal action, and a MIC_100_ of 250 µg/mL on *E. faecalis*. Instead, (*R*)-phomalactone (**1**) exerted growth-inhibitory effects on the Gram-negative strains *A. baumannii* and *P. aeruginosa*, with MIC_100_ values of 125 µg/mL and 500 µg/mL, respectively. Finally, neither the crude extract nor the pure compounds **1**–**3** affected *Candida* growth. These results are in agreement with the very little data available so far in the literature regarding the antibacterial properties of phomalactone. To the best of our knowledge, only K. P. Ramesha et al. (2020) had previously highlighted the antibacterial potential of phomalactone derived from the endophyte *Nigrospora sphaerica*, which showed a broad spectrum of antimicrobial activity against human and phytopathogenic bacteria and fungi [32].

## 3. Materials and Methods

### 3.1. General Experimental Procedure

Optical rotations were measured in CHCl_3_ on a Jasco DIP 370 digital polarimeter (Jasco, Lecco, Italy). NMR experiments were recorded at the ICB-NMR Service Centre on a Bruker (Karlshrue, Germany) Avance III HD 400 MHz spectrometer equipped with a CryoProbe Prodigy and on a Bruker DRX-600 operating at 600 MHz, using an inverse TCI CryoProbe fitted with a gradient along the *Z*-axis. Chemical shift values are reported in ppm and referenced to internal signals of residual protons (CDCl_3_, δ 7.26 for H-atom, δ 77.0 for carbon). High-Resolution Electron Spray Ionization Mass Spectra (HRESIMS) were acquired on a Q-Exactive hybrid quadrupole-orbitrap mass spectrometer (Thermo Scientific, San Jose, CA, USA). Analytical and preparative TLC were performed on precoated SiO_2_ plates (Merck Kieselgel 60 F254, 0.25 and 0.5 mm) (Merck, Milan, Italy), with detection provided by UV light (254 nm) and by spraying with CeSO_4_ reagent followed by heating (120 °C). SiO_2_ column chromatography was performed using Merck Kieselgel 60 powder (0.063–0.200 mm) (Merck, Milan, Italy).

### 3.2. Fungal Identification

For molecular identification, the fungal strain was subjected to internal transcribed spacer regions (ITS) and *glyceraldehyde-3-phosphate dehydrogenase* (*gpd*) gene sequence analysis. The strain was grown on Potato dextrose agar (PDA, Oxoid Ltd., Basingstoke, UK) for 5 days at 25 °C and the mycelium was collected, frozen at −20 °C overnight, and extracted using Wizard^®^ Magnetic DNA Purification System for Food Kit (Promega, Madison, WI, USA). Extracted DNA was analyzed by PCR using DreamTaq™ Hot Start DNA Polymerase (Thermo Fisher Scientific, Santa Clara, CA, USA) and the following primer pairs, according to the manufacturer’s instructions: for internal transcribed spaces regions, ITS4 (TCCTCCGCTTATTGATATGC) and ITS5 (GGAAGTAAAAGTCGTAACAAGG) [33], and for *glyceraldehyde-3-phosphate dehydrogenase*, *Gpd1* (CAACGGCTTCGGTCGCATTG), and *Gpd2* (GCCAAGCAGTTGGTTGTGC) [34] were used. Each PCR reaction was performed in SimpliAmp™ Thermal Cycler (Applied Biosystems, Foster City, CA, USA) and the PCR reaction was carried out with the following conditions: initiation for 3 min at 95 °C, 33 cycles of denaturation for 30 s at 95 °C, annealing for 30 s at 55 °C for ITS and 59 °C for *gpd*, extension for 1 min at 72 °C, and a final extension for 5 min at 72 °C. To determine DNA concentration (ng/μL) and purity (A260/A280 and A260/A230 ratio), a NanoDrop OneC (Thermo Fisher Scientific, USA) was used and a gel electrophoresis on 1.5% (*w*/*v*) agarose gel stained with GelRed (Biotium, Fremont, CA, USA) was performed to evaluate DNA integrity. The PCR products were purified with the ExoSAP-IT™ Express PCR Product Cleanup (Thermo Fisher Scientific, Santa Clara, CA, USA) and the purified products were sequenced with the BigDye™ Terminator v3.1 Cycle Sequencing Kit (Applied Biosystems, Foster City, CA, USA), purified by filtration through Sephadex G-50 (Sigma-Aldrich, Saint Louis, MO, USA), and sequenced in SeqStudio™ 8 Flex Series Genetic Analyzer (Applied Biosystems, Foster City, CA, USA). The nucleotide sequences obtained, after cleaning and alignment, were compared with those of strains stored in the NCBI nucleotide database using the research tool BLAST-N (https://blast.ncbi.nlm.nih.gov accessed on 4 April 2025), and the identification was confirmed based on the highest score of alignment and by percentage of identity > 99%.

### 3.3. Fungal Growth

The fungus was routinely grown in Petri dishes on PDA medium. Pieces of mycelium taken aseptically from these plates were used to seed a defined liquid medium, M1D [35]. M1D medium consisted of (mg/L) sucrose 20,000, MgSO_4_ × 7 H_2_O 360, Na_2_SO_4_ 200, Ca(NO_3_)_2_ × 4 H_2_O 200, KNO_3_ 80, KCl 65, NaH,PO, × H_2_O 16.5, MnSO_4_ × 4 H_2_O 4.5, ferric citrate 2.0, ZnSO_4_ × 7 H_2_O 1.5, H_3_BO_3_ 1.5, KI 0.75, 2,4-dichlorophenoxyacetic acid 0.5, nicotinic acid 0.5, pyridoxin 0.1, and thiamine 0.1. For the stirred condition, Erlenmeyer flasks containing 400 mL of medium were seeded and incubated at 25 °C at 120 rpm in an orbital shaker for 5 days. The mycelia were kept away by filtering the culture filtrates through two layers of cheesecloth to reduce the fungal biomass and further using 45 μm Millipore filters [36]. After separation from the mycelium, the culture filtrates obtained from the static growth conditions (ca. 1.7 L) were lyophilized; roux bottles containing 200 mL of medium were seeded and incubated at 25 °C in a camera for 4 weeks. The same protocol was applied on the culture filtrate obtained from stirring (ca. 1.5 L), which after the removal of the mycelium, was as well lyophilized.

### 3.4. Extraction and Purification of Bioactive Metabolites

The lyophilized fungal culture filtrates (1.7 L) were dissolved in distilled water (300 mL, final pH 4.0) and extracted with EtOAc (3 × 250 mL). The organic extracts were combined and evaporated under reduced pressure to give a brown oil residue (1.641 g). The residual aqueous phase was acidified to pH 2 with formic acid (HCOOH) and extracted in the same condition, yielding a brown oily residue (402.5 mg). The corresponding lyophilized mycelia (7.2 g) was extracted with EtOAc (300 mL) under stirring. After 12 h, the suspension was centrifuged (7000 rpm at 10 °C) and the supernatant evaporated under reduced pressure to give an oily residue (306.5 mg). The same procedure was applied to both the lyophilized fungal culture filtrates obtained in static conditions (1.5 L) and corresponding mycelia (9.1 g). This yielded three brown oily extracts from the original culture, the acidified culture, and the mycelia, with weights of 891.8 mg, 338.2 mg, and 391.8 mg, respectively. The comparison of all the extracts by TLC eluted with different solvents system (CHCl_3_/*i*-PrOH 95:5, CHCl_3_/MeOH 9:1, petroleum ether/diethyl ether 1:1) showed an identical metabolic profile. The organic extract of mycelia was not worked. Therefore, the organic extracts obtained from stirred and static culture filtrates were combined and the resulting residue (2.8 g) was purified on silica gel column eluted with CHCl_3_/i-PrOH (95:5), yielding ten homogeneous fraction groups (3/F1-3/F10). Fraction 3/F4 (0.726 g) was further purified in the same condition, affording four groups of homogeneous subfractions (F4/1-F4/4). Subfractions F4/1 and F4/3 (34.2 and 379.4 mg, respectively) appeared to be homogeneous compounds and, after NMR analysis, were identified as dihydrophomalactone (**2**, R*f* 0.50, CHCl_3_/*i*-PrOH 95:5) and phomalactone (**1**, R*f* 0.40, CHCl_3_/i-PrOH 95:5), respectively. Fraction F3/5 (250.3 mg) was further purified on silica gel and eluted with CHCl_3_/i-PrOH (95:5), giving twelve groups of homogeneous subfractions (F5/1-F5/12). Subfraction F5/9 (71.9 mg) was a homogeneous compound, identified after NMR analysis as (–)-asperpentyn (**3**, R*f* 0.35, CHCl_3_/*i*-PrOH 95:5).

### 3.5. Phytotoxic Bioassays

#### 3.5.1. Leaf Puncture Assay

Droplets (30 µL) of the culture filtrates were applied to detached leaves of mauve (*Calluna vulgaris* L.), vitriol grass (*Parietaria officinalis* L.), marigold (*Calendula officinale* L.), perennial sowthistle (*Sonchus arvensis* L.), and hemp (*Cannabis sativa* L.) previously punctured with a needle. Three replications were used for each plant species tested. Leaves were kept in a moistened chamber under continuous fluorescent light at 25 °C. The eventual appearance of symptoms, consisting of circular necrosis, was observed 3 days after droplet application. Control treatments were carried out by applying droplets of a defined mineral medium.

#### 3.5.2. Tomato Cutting Assay

Each sample was dissolved in a minute amount of MeOH and brought up to the required concentration with distilled H_2_O. The final concentration of MeOH was 5%, also used as a negative control. The phytotoxic activity of compounds **1**–**3** was tested at 1 mg/mL on tomato (*Solanum lycopersicum* L.) cuttings using the absorption method previously reported [37,38]. Ophiobolin A, at the same concentration, was used as a positive control. The assays were performed in triplicate (see Figure S1 in Supporting Materials).

### 3.6. Test Strains and Culture Conditions

The antimicrobial activity of the crude organic extract of fungal culture filtrates and that of compounds **1**–**3** was assayed on different bacterial reference strains, both Gram-positive and Gram-negative, and on a yeast reference strain belonging to the American Type Culture Collection (ATCC) (Rockville, MD, USA). More specifically, the Gram-positive test strains were *Staphylococcus aureus* ATCC 6538, *methicillin-resistant Staphylococcus aureus* (MRSA) ATCC 43300, and *Enterococcus faecalis* ATCC 29212 and the Gram-negative test strains were *Pseudomonas aeruginosa* ATCC 27853 and *Acinetobacter baumannii* ATCC BAA747; the yeast test strain was *azole-resistant Candida albicans* ATCC 10231. All strains were stored as 15% (*v*/*v*) glycerol stocks at −80 °C. Before each experiment, bacterial cells were sub-cultured from the stocks onto tryptic soy agar (TSA; Becton Dickinson, Franklin Lakes, NJ, USA) plates at 37 °C for 24 h, while yeast cells were sub-cultured from the stocks onto sabouraud dextrose agar (SAB; Becton Dickinson) plates at 37 °C for 24/48 h. The identification of the test strains was performed by the Vitek II (bioMérieux, Marcy-l’Étoile, France) system and MS MALDI-TOF (Bruker Daltonics, Bremen, Germany).

### 3.7. Antimicrobial Assay

The antimicrobial activity was assayed by a standard broth micro-dilution method in 96-well polystyrene plates. First, 0,5 McFarland cell suspensions in Brain Heart Infusion for the bacterial strains (1.5–5 × 10^8^ colony-forming unit [CFU]/mL) and Sabouraud dextrose broth for the *Candida* strain (4 × 10^6^ CFU/mL per well) were prepared. For each strain, aliquots (100 μL) of 1:100 dilutions in fresh media were dispensed into each well. The activity of each sample was tested by adding 100 µL of two-fold dilutions, starting from 1 mg/mL of solution, to the wells. The wells without substances were used as a positive growth control. Conventional antibiotics, selected depending on antibiotic susceptibility profiles of the test strains, were included as the control; colistin (ranged from 0.2 to 12.5 µg/mL) was used for Gram-negative strains and teicoplanin (ranged from 0.5 to 4 µg/mL) for Gram-positive strains. Amphotericin B (ranged from 0.12 to 2 μg mL) was used for the yeast strain. Plates were incubated at 37 °C for 19 h under shaking (300 rpm). Then, the medium turbidity was measured by a spectrophotometer at 595 nm (Bio-Rad Laboratories Srl, Hercules, CA, USA). The antimicrobial activity was expressed as a percentage of microbial growth inhibition. The minimal inhibitory concentration (MIC) was defined as the lowest concentration of sample that caused no visible microbial growth in the wells. The extract and each metabolite were tested in triplicate and each experiment was performed twice. To be sure that the 2% of dimethyl sulfoxide (DMSO; Sigma-Aldrich, St. Louis, MO, USA) in the 2× stock solutions of the samples did not affect the microbial growth, the effect of serial dilutions of DMSO in fresh media, starting from 1%, on the growth of the test strains was separately tested.

### 3.8. Antifungal Bioassay

The antifungal activity of mycelia extract, culture filtrates, and three purified compounds [F4/3 (**1**); F4/1 (**2**); F49–50 (**3**)] of *C. inequalis* were assayed against four different phytopathogenic fungi: *Macrophomina phaseolina*, *Botrytis cinerea*, *Alternaria alternata*, and *Septoria nodorum*, present in the collection of the University of Naples Federico II, Department of Biology (Italy) [38,39,40]. Briefly, the mycelial plugs (4-day-old culture) of 5 mm diameter of each fungus were located in the center of potato dextrose agar (PDA) (Difco, Mansoura, Lebanon) plates. The mycelia extract and culture filtrates were dissolved in 5% *v*/*v* MeOH, and amounts of 100, 50, and 10 μg total in 20 μL (corresponding to 5 μg/μL, 2.5 μg/μL, and 0.5 μg/μL) were applied to the tops of the mycelial plugs. The three purified compounds (**1**–**3**) were tested at 2.5 μg/μL and 20 μL of 5% MeOH was applied to the tops of the mycelial plugs and used as a negative control. The solvent was allowed to evaporate in a laminar flow cabinet, and the plates were incubated at 28 °C for 5–7 days. The percentage of inhibition of the fungal growth was calculated using the following formula % = [(Rc − Ri)/Rc ] × 100, where Rc is the radial growth of the test pathogen in the control plates (mm) and Ri is the radial growth of the test pathogen in the presence of compounds tested (mm). The experiment was performed in triplicate and data are presented as the mean ± standard deviation.

## 4. Conclusions

The chemical study carried out on the culture filtrate of a strain of *Curvularia inaequalis*, an endophytic fungus newly isolated from the Iranian medicinal plant *Echium khuzistanicum*, led to the isolation and identification of three bioactive metabolites: (*R*)-phomalactone (**1**), catenioblin A (**2**), and (–)-asperpentyn (**3**). The results from the biological assays confirm the promising properties of (*R*)-phomalactone (**1**), revealing its broad-spectrum antimicrobial effects. New data shows its potency against a range of human pathogens, such as methicillin-resistant *S. aureus* (MRSA), in addition to its efficacy against several important plant fungal pathogens. This broad efficacy is particularly noteworthy given the growing threat of microbial resistance in both medicine and agriculture. The bioactivity observed for phomalactone paves the way for additional investigation, including structural modifications and toxicological studies, to assess its potential as a therapeutic agent or sustainable agrochemical. Furthermore, (–)-asperpentyn (**3**) also demonstrated robust and selective antifungal activity against phytopathogens, suggesting its viability as a targeted natural fungicide. In conclusion, our findings highlight once again that the exploration of plant–endophyte associations, particularly those from underexplored medicinal flora, represents a strategic tool for the discovery of new sources of bioactive compounds.

## Figures and Tables

**Table 1:** MIC_≥90_ (μg/mL) values of active compounds against tested strains.

Tested Strains	Crude Organic Extract	(+)-Phomalactone	Teicoplanin	Colistin	Amphotericin B
*S. aureus* (ATCC 6538)	250	62	0.5	^a^	^a^
*MRSA* (ATCC 43300)	250	62	1	^a^	^a^
*E. faecalis* (ATCC 29212)	1000	250	0.5	^a^	^a^
*P. aeruginosa* (ATCC 27853)	1000	500	^a^	1	^a^
*A. baumanii* (ATCC BAA747)	1000	125	^a^	0.5	^a^
*C. albicans* (ATCC 10231)	1000	>500	^a^	^a^	0.25

^a^ not applicable.

## Data Availability

Data are contained within the article and Supplementary Materials.

## Supplementary Materials

The following supporting information can be downloaded at https://www.mdpi.com/article/10.3390/molecules30193870/s1: Figure S1: Tomato cutting assay; NMR spectroscopy and HR-ESI-MS; Figure S2: ^1^H NMR spectrum of (*R*)-phomalactone (**1**); Figure S3: COSY spectrum of (*R*)-phomalactone (**1**); Figure S4: *ed*-HSQC spectrum of (*R*)-phomalactone (**1**); Figure S5: HMBC spectrum of (*R*)-phomalactone (**1**); Figure S6: ^13^C NMR spectrum of (*R*)-phomalactone (**1**); Figure S7: HR-(+)-ESIMS spectrum of (*R*)-phomalactone (**1**); Figure S8: ^1^H NMR spectrum of catenioblin A (**2**); Figure S9: COSY spectrum of catenioblin A (**2**); Figure S10: *ed*-HSQC spectrum of catenioblin A (**2**); Figure S11: HMBC spectrum of catenioblin A (**2**); Figure S12: ^13^C NMR spectrum of catenioblin A (**2**); Figure S13: HR-(+)-ESIMS spectrum of catenioblin A (**2**); Figure S14: ^1^H NMR spectrum of (–)-asperpentyn (**3**); Figure S15: COSY spectrum of (–)-asperpentyn (**3**); Figure S16: *ed*-HSQC spectrum of (–)-asperpentyn (**3**); Figure S17: HMBC spectrum of (–)-asperpentyn (**3**); Figure S18: ^13^C NMR spectrum of (–)-asperpentyn (**3**); Figure S19: HR-(-)-ESIMS spectrum of (–)-asperpentyn (**3**).
