# Supplementary material for: Extraction and Identification of the Bioactive Metabolites Produced by Curvularia inaequalis, an Endophytic Fungus Collected in Iran from Echium khuzistanicum Mozaff
Source: Molecules. 2025 Sep 24;30(19):3870. doi: 10.3390/molecules30193870 (PMC12526211; doi:10.3390/molecules30193870)
Supplement: Supplementary file 1 [file molecules-30-03870-s001.zip › molecules-3805550-supplementary.pdf]

Supplementary Materials

On the bioactive metabolites produced by *Curvularia inaequalis*, an endophytic fungus collected in Iran from *Echium khuzistanicum* Mozaff

Maryam Besharati,[1,2] Maria Letizia Ciavatta,[1,*] Marianna Carbone,[1] Nadia Cacciapuoti,[1] Martina Aversa,[3] Emanuela Roscetto,[3] Stefany Castaldi,[4] Giancarlo Perrone,[5] Angela Boari,[5] Katia Gialluisi,[5] Maria Rosaria Catania,[3] S. Ali Moosawi-Jorf,[2] and Antonio Evidente[1]

[1] Institute of Biomolecular Chemistry (ICB), National Research Council (CNR), Viale campi Flegrei 34, 80078 Pozzuoli, Italy; besharatimaryam513@yahoo.com (M.B.); marialetizia.ciavatta@cnr.it (M.L.C.); marianna.carbone@cnr.it (M.C.); evidente@unina.it (A.E.).
[2] Tarbiat Modares University, Faculty of Agriculture, Intersection of Chamran and Jalal Ale-Ahmad Highway, Tehran, Iran; besharatimaryam513@yahoo.com (M.B.); moosawijorf@modares.ac.ir (S.A.M.-J.)
[3] Department of Molecular Medicine and Medical Biotechnologies, University of Naples Federico II, Via Pansini 5, 80131 Napoli, Italy; martina.aversa@unina.it (M.A.); emanuela.roscetto@unina.it (E.M.); mariarosaria.catania@unina.it (M.R.C.)
[4] Department of Biology, University of Naples Federico II, Complesso Universitario Monte S. Angelo,Via Cintia 4, 80126 Napoli, Italy; stefany.castaldi@unina.it (S.C.)
[5] Institute of Sciences of Food Production, National Research Council (CNR) Via Amendola 122/O, 70125 Bari, Italy; Giancarlo.perrone@cnr.it (G.P.); angela.boari@cnr.it (A.B.);

* Correspondence: evidente@unina.it

Content:

Figure S1.	Figure SM1. Tomato cutting assay
Figure S2.	^{1}H NMR spectrum of (*R*)-phomalactone (**1**) (Bruker 600MHz, $CDCl_3$)
Figure S3	COSY spectrum of (*R*)-phomalactone (**1**) (Bruker 600MHz, $CDCl_3$)
Figure S4	*ed*-HSQC spectrum of (*R*)-phomalactone (**1**) (Bruker 600MHz, $CDCl_3$)
Figure S5	HMBC spectrum of (*R*)-phomalactone (**1**) (Bruker 600MHz, $CDCl_3$)
Figure S6	^{13}C NMR spectrum of (*R*)-phomalactone (**1**) (Bruker 150 MHz, $CDCl_3$)
Figure S7	HR-(+)-ESIMS spectrum of (*R*)-phomalactone (**1**)
Figure S8	^{1}H NMR spectrum of catenioblin A (**2**) (Bruker 400MHz, $CDCl_3$)
Figure S9	COSY spectrum of catenioblin A (**2**) (Bruker 400MHz, $CDCl_3$)
Figure S10	*ed*-HSQC spectrum of catenioblin A (**2**) (Bruker 400MHz, $CDCl_3$)
Figure S11	HMBC spectrum of catenioblin A (**2**) (Bruker 400MHz, $CDCl_3$)
Figure S12	^{13}C NMR spectrum of catenioblin A (**2**) (Bruker 100 MHz, $CDCl_3$)
Figure S13	HR-(+)-ESIMS spectrum of catenioblin A (**2**)
Figure S14	^{1}H NMR spectrum of (–)-asperpentyn (**3**) (Bruker 400MHz, $CDCl_3$)
Figure S15	COSY spectrum of (–)-asperpentyn (**3**) (Bruker 400MHz, $CDCl_3$)
Figure S16	*ed*-HSQC spectrum of (–)-asperpentyn (**3**) (Bruker 400MHz, $CDCl_3$)
Figure S17	HMBC spectrum of (–)-asperpentyn (**3**) (Bruker 400MHz, $CDCl_3$)
Figure S18	^{13}C NMR spectrum of (–)-asperpentyn (**3**) (Bruker 100 MHz, $CDCl_3$)
Figure S19	HR-(-)-ESIMS spectrum of (–)-asperpentyn (**3**)

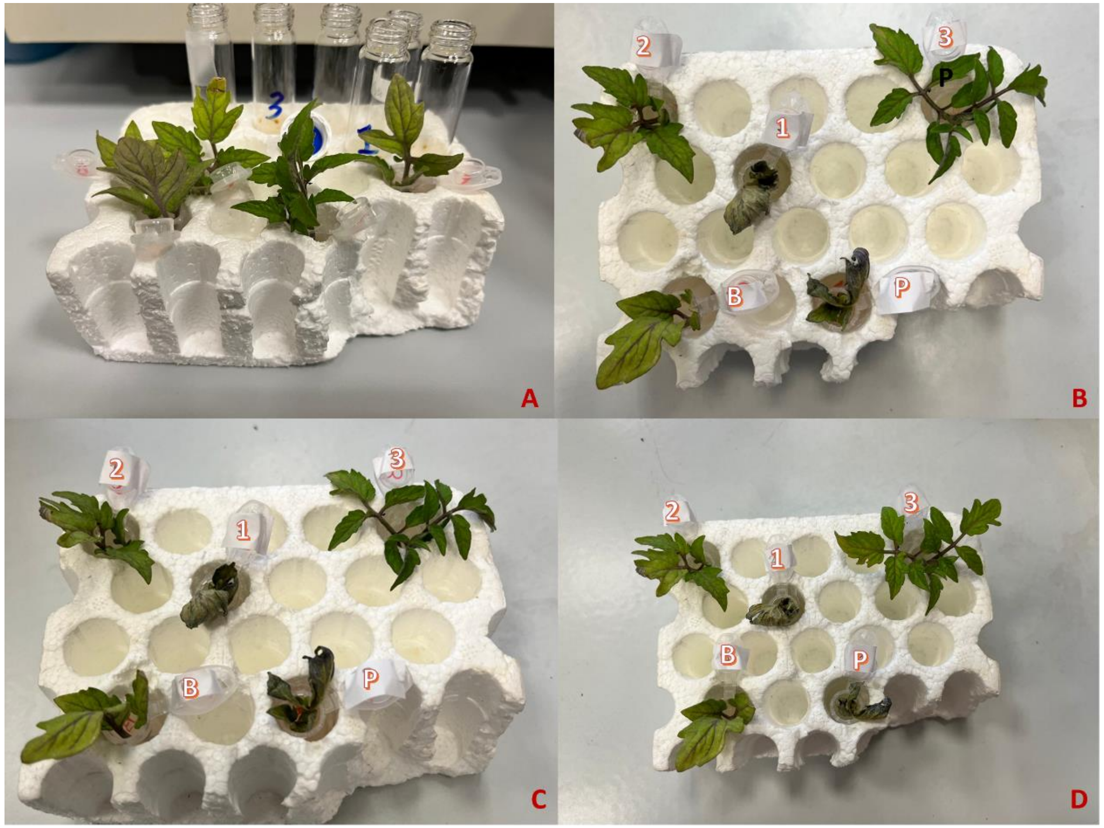

Tomato cuttings under different treatment conditions and over time. In figure A tomato cutting at t0; in B tomato cutting after 24h treatment with pure compounds (*R*)-phomalactone (**1**), catenioblin A (**2**), (-)asperpentyn (**3**), only vehicle (**B**), and with ophiobolin A as positive control (**P**); in C tomato cutting after 48 h treatment; in D tomato cuttings at 72 hours, 24 hours after all treatment solutions were replaced with water at the 48-hour mark. The assay was performed in triplicates.

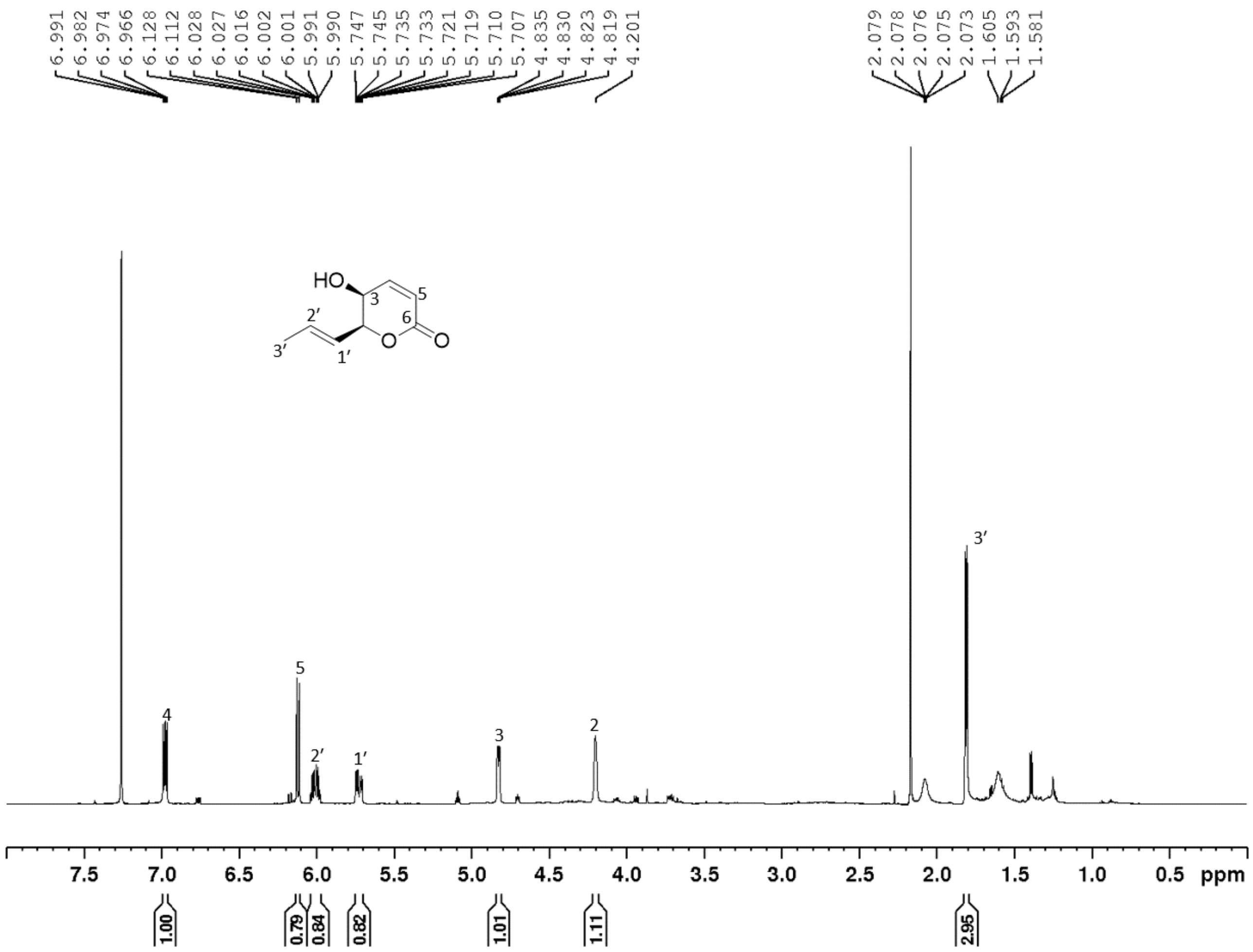

^{1}H NMR spectrum of (*R*)-phomalactone (**1**) (Bruker 600MHz, $CDCl_3$)

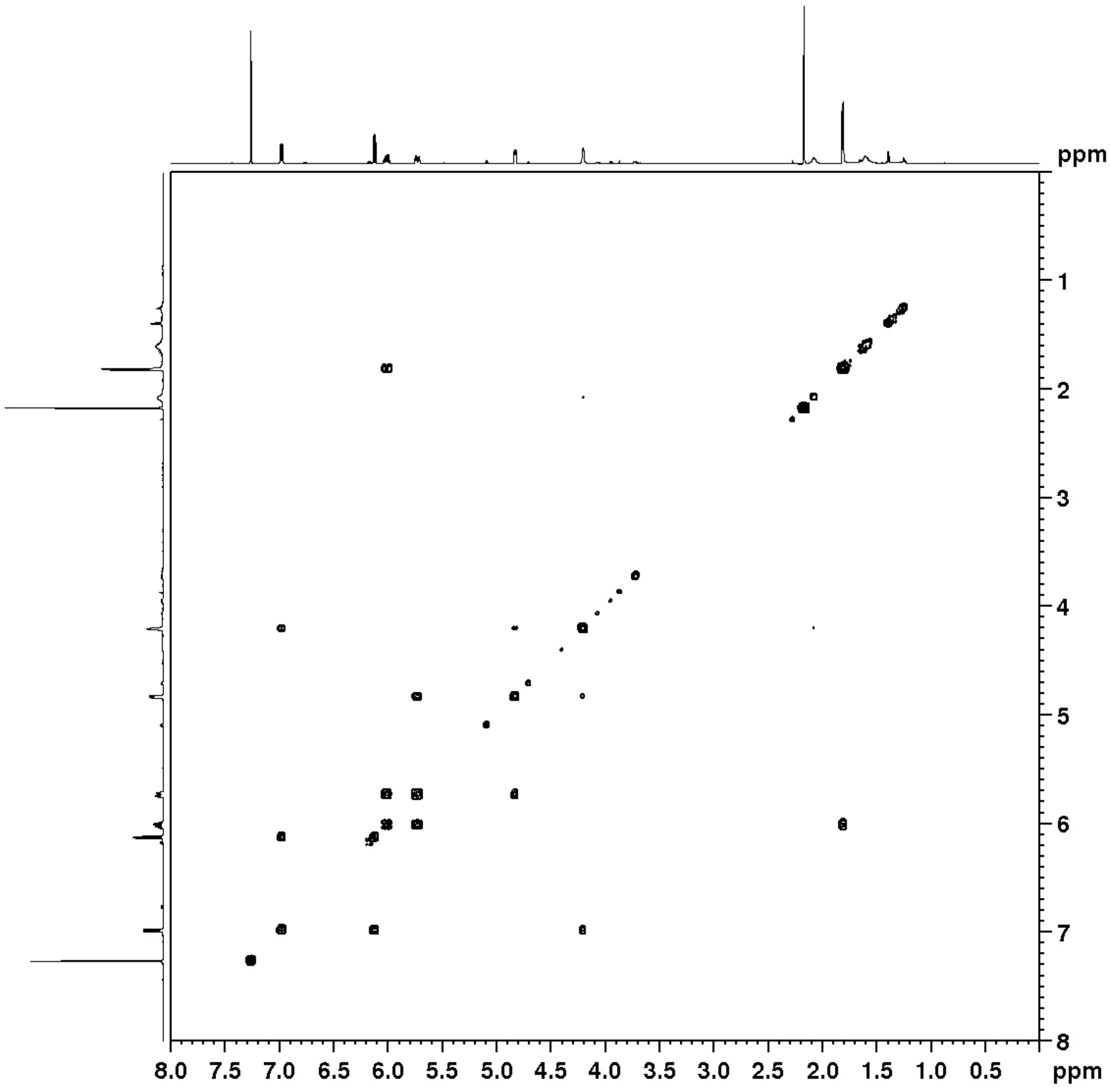

COSY spectrum of (*R*)-phomalactone (**1**) (Bruker 600MHz, $CDCl_3$)

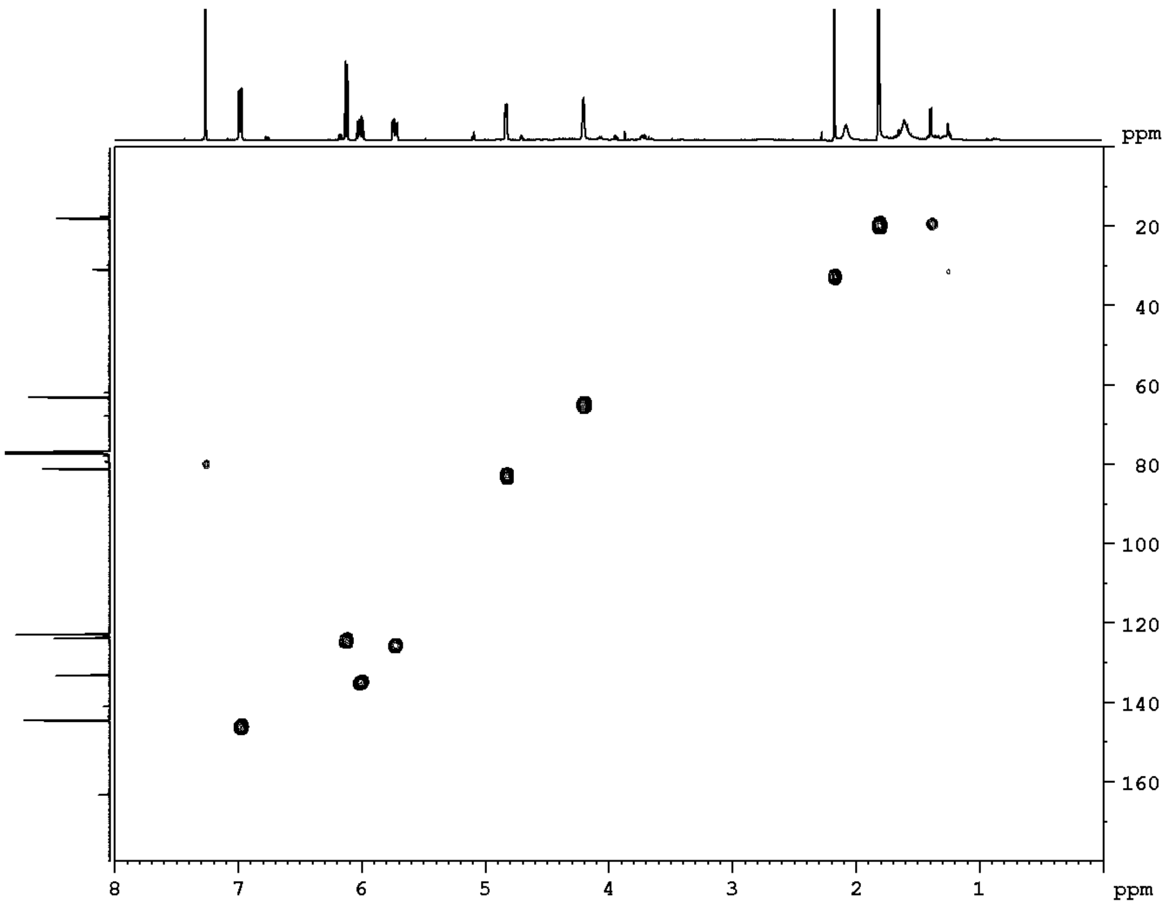

ed-HSQC spectrum of (*R*)-phomalactone (**1**) (Bruker 600MHz, $CDCl_3$)

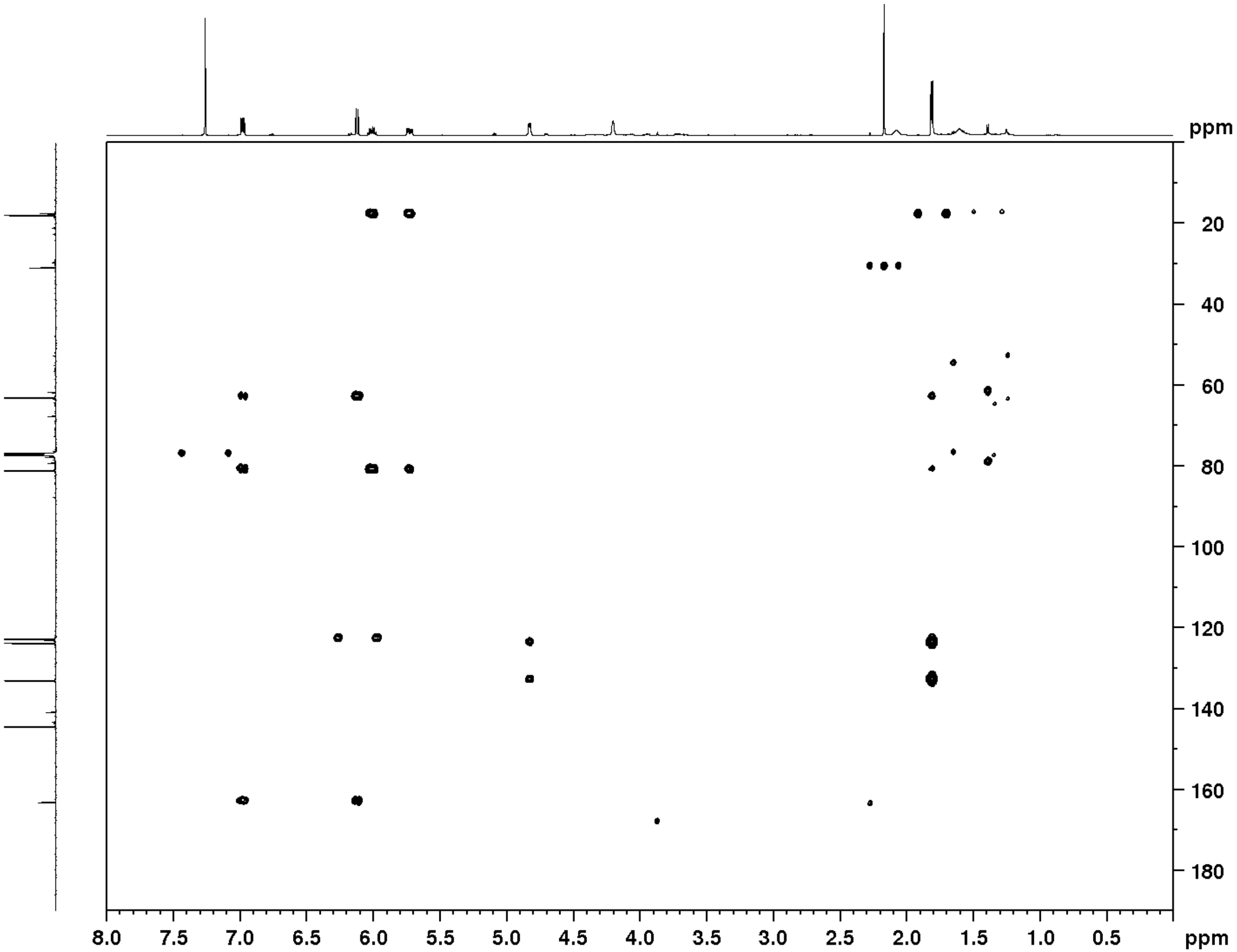

HMBC spectrum of (*R*)-phomalactone (**1**) (Bruker 600MHz, $CDCl_3$)

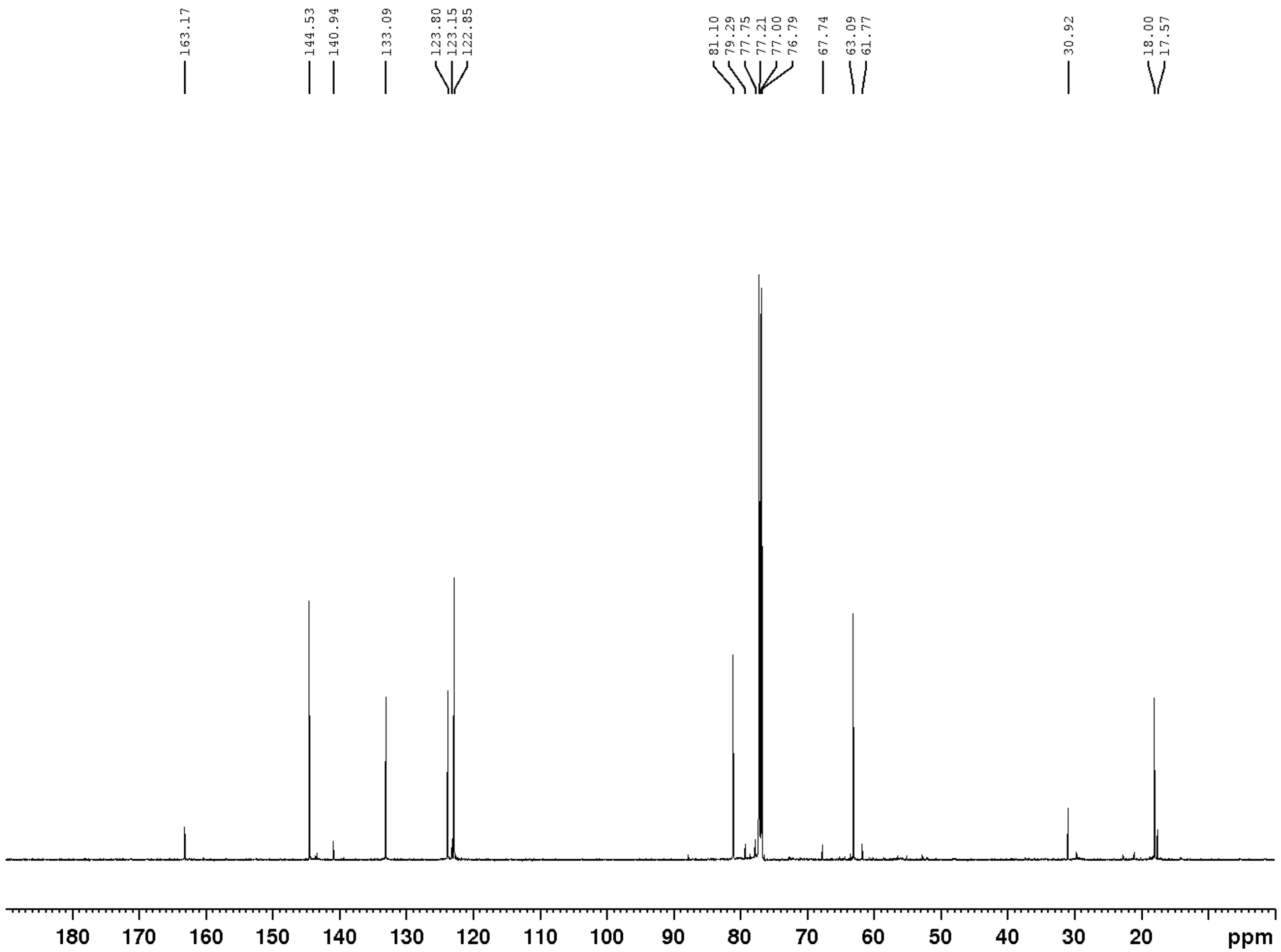

^{13}C NMR spectrum of (*R*)-phomalactone (**1**) (Bruker 150 MHz, $CDCl_3$)

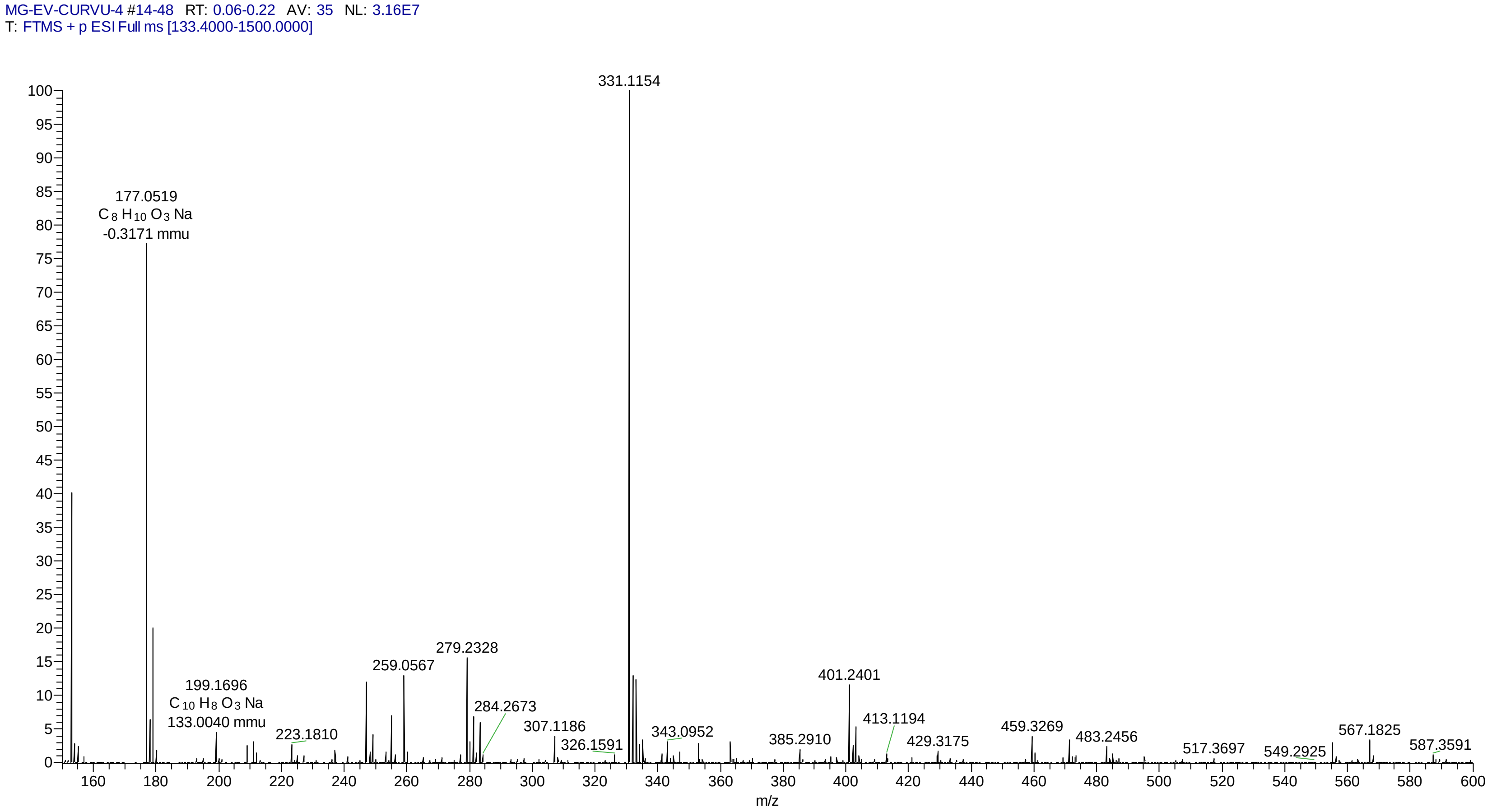

HR-(+)-ESIMS spectrum of (*R*)-phomalactone (**1**)

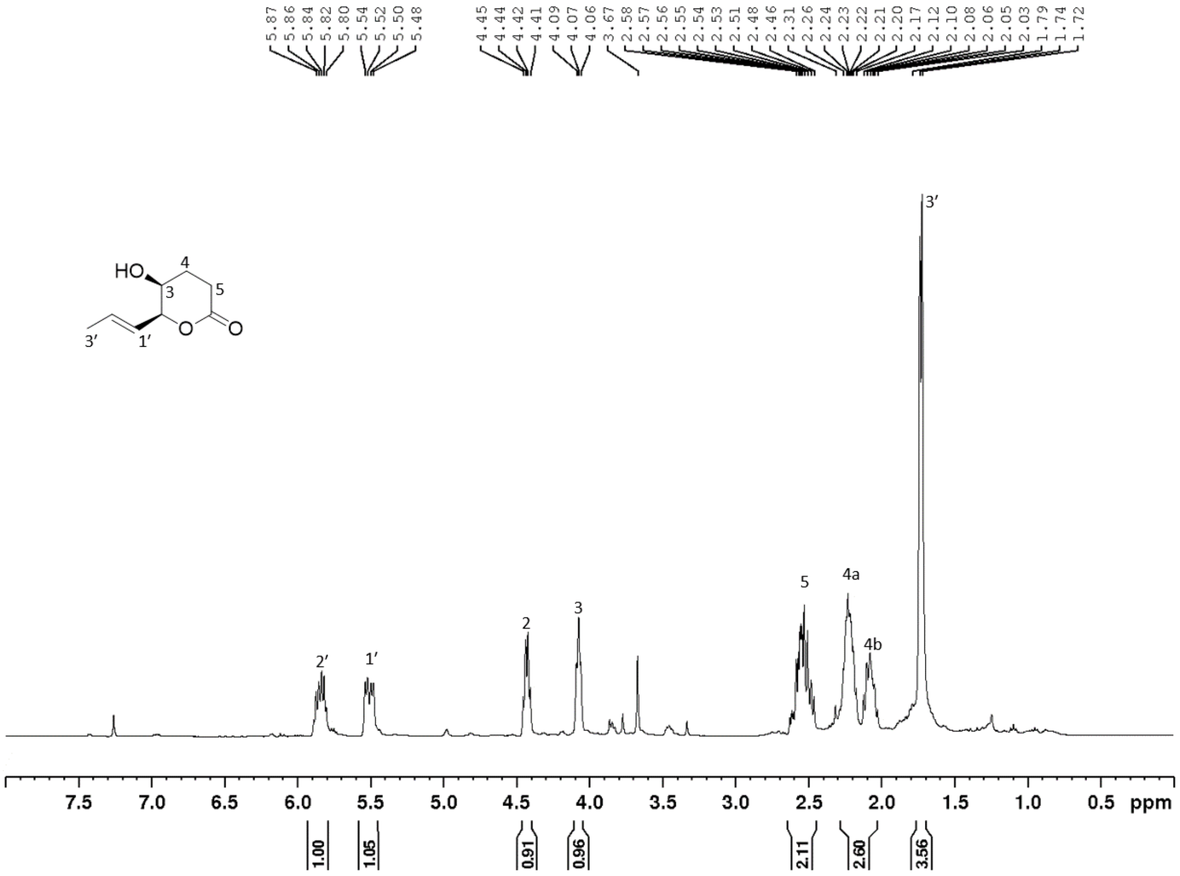

^{1}H NMR spectrum of catenioblin A (**2**) (Bruker 400MHz, $CDCl_3$)

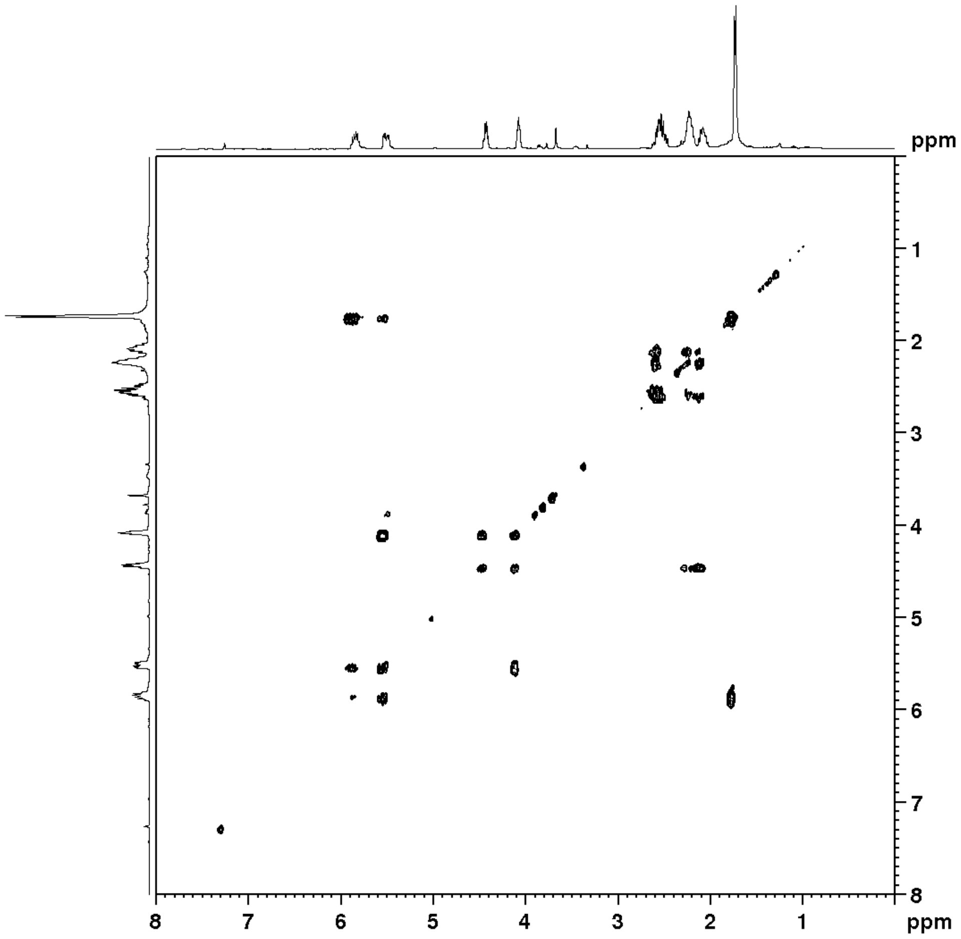

COSY spectrum of catenioblin A (**2**) (Bruker 400MHz, $CDCl_3$)

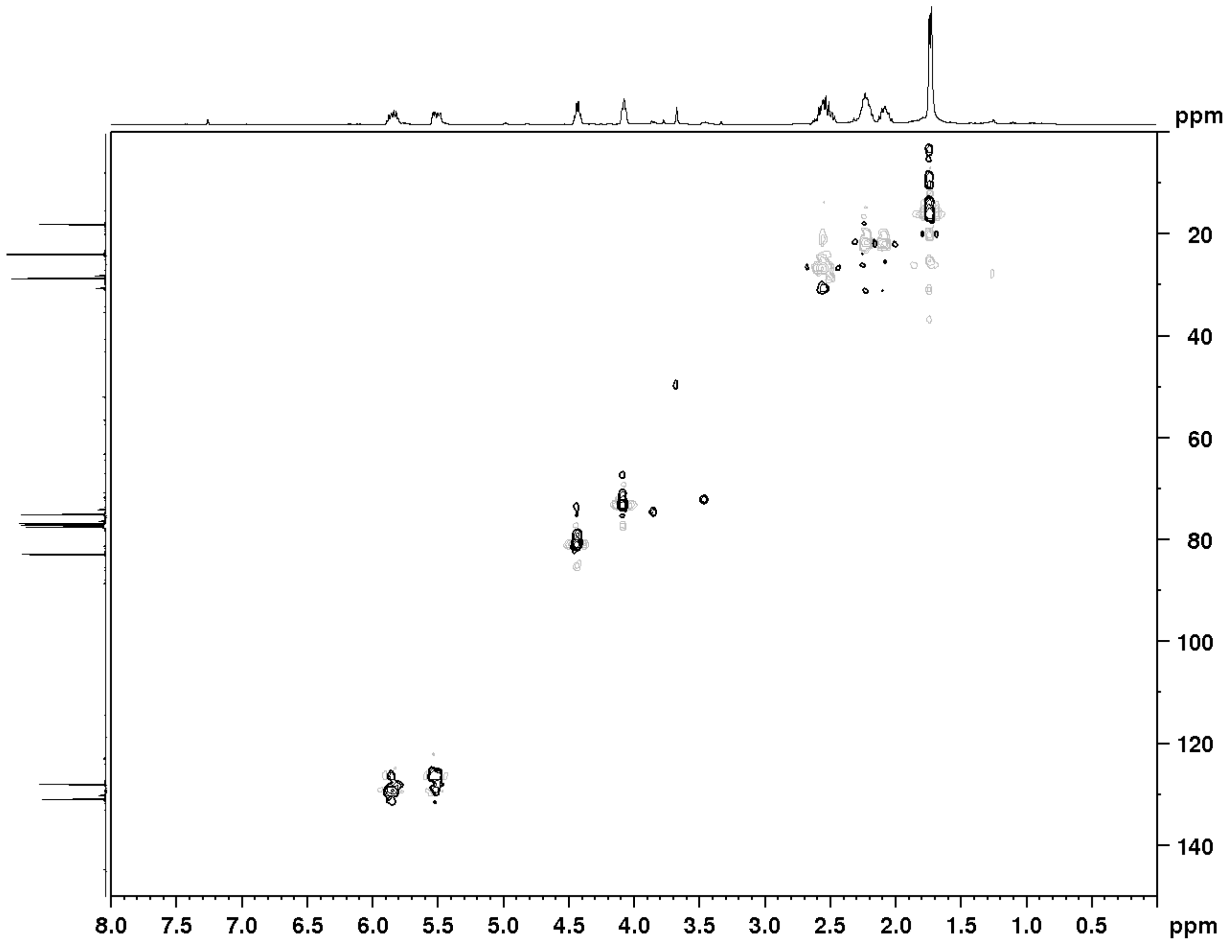

ed-HSQC spectrum of catenioblin A (**2**) (Bruker 400MHz, $CDCl_3$)

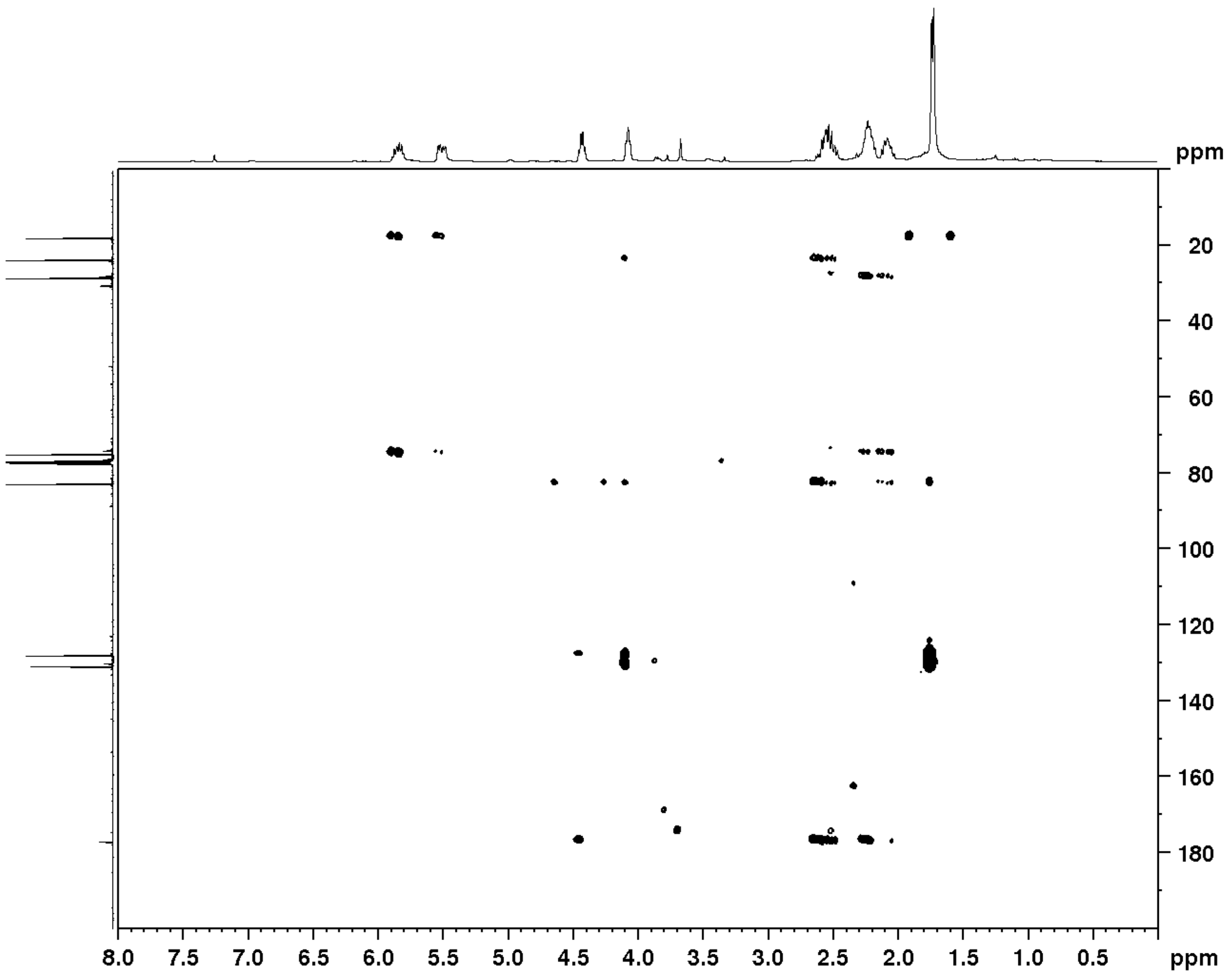

HMBC spectrum of catenioblin A (**2**) (Bruker 400MHz, $CDCl_3$)

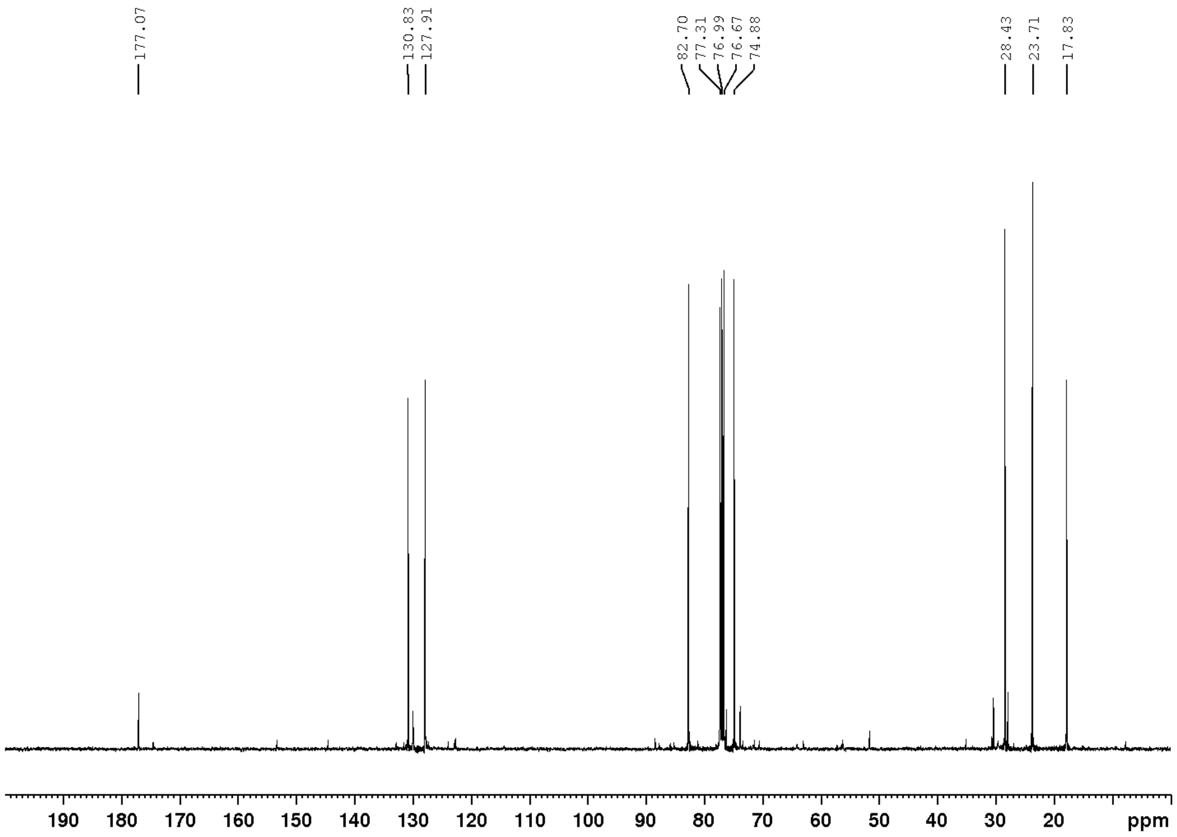

^{13}C NMR spectrum of catenioblin A (**2**) (Bruker 100 MHz, $CDCl_3$)

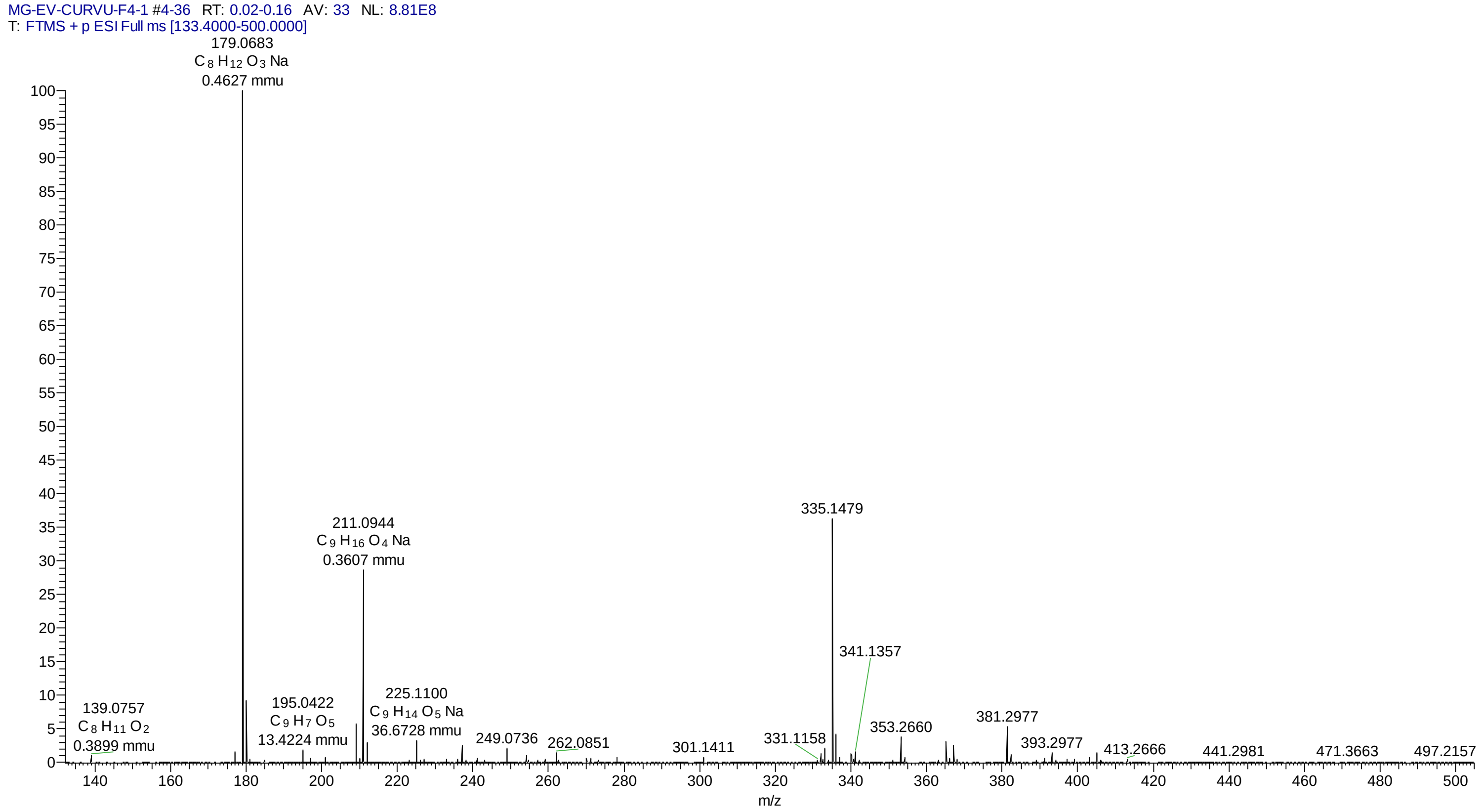

HR-(+)-ESIMS spectrum of catenioblin A (**2**)

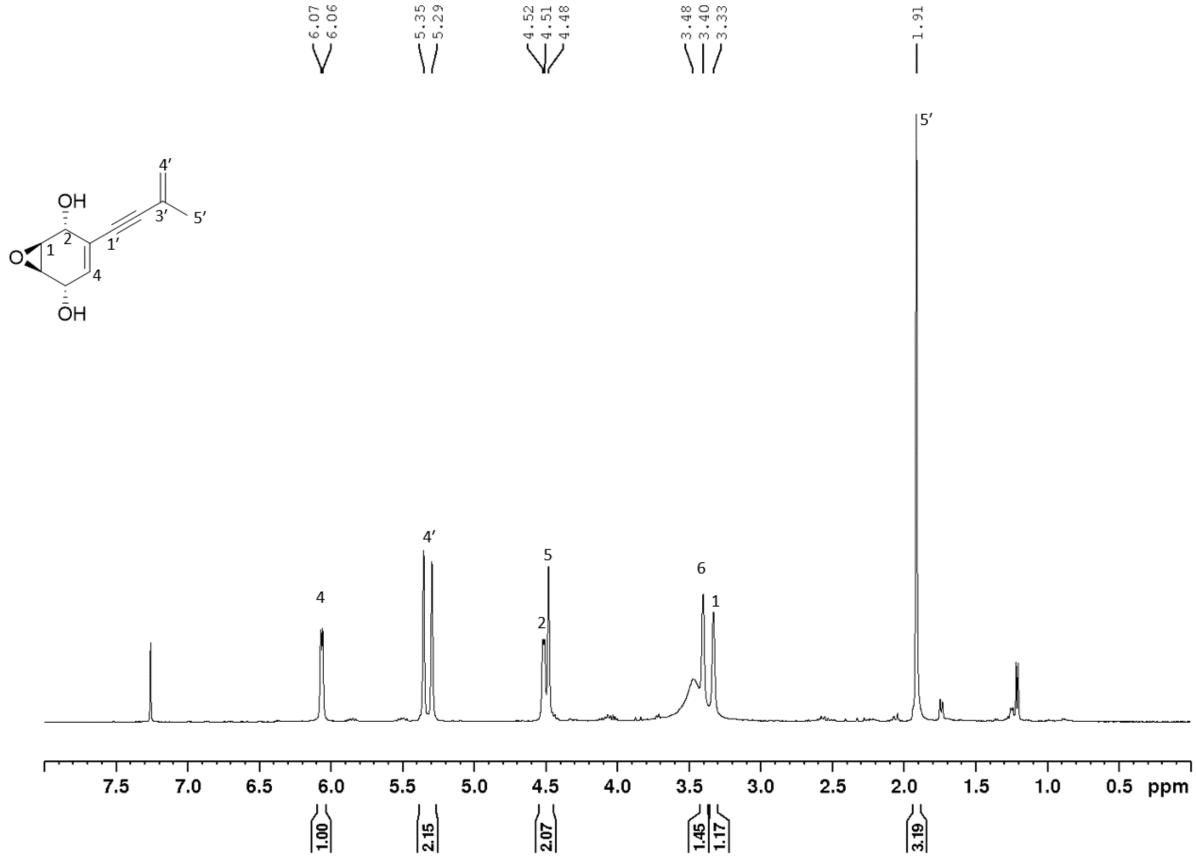

^{1}H NMR spectrum of (–)-asperpentyn (**3**) (Bruker 400MHz, $CDCl_3$)

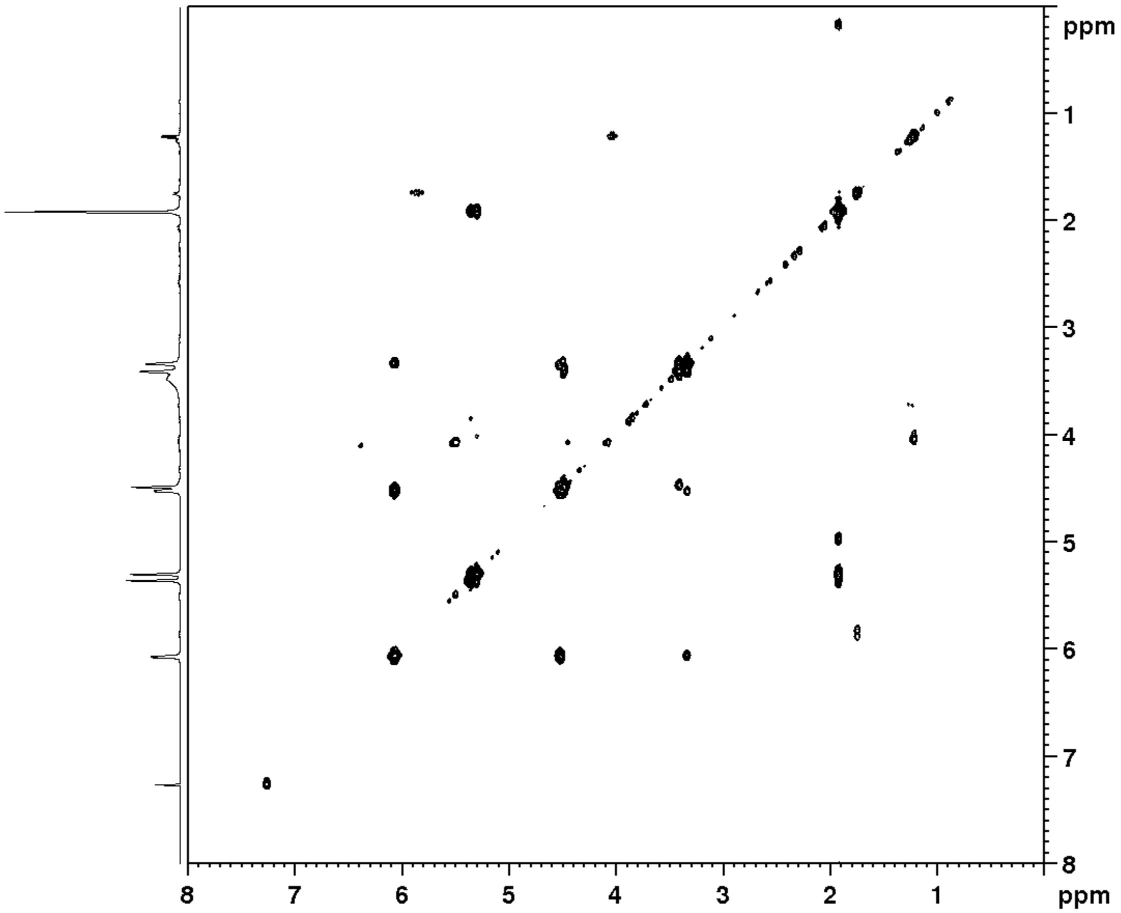

COSY spectrum of (–)-asperpentyn (**3**) (Bruker 400MHz, $CDCl_3$)

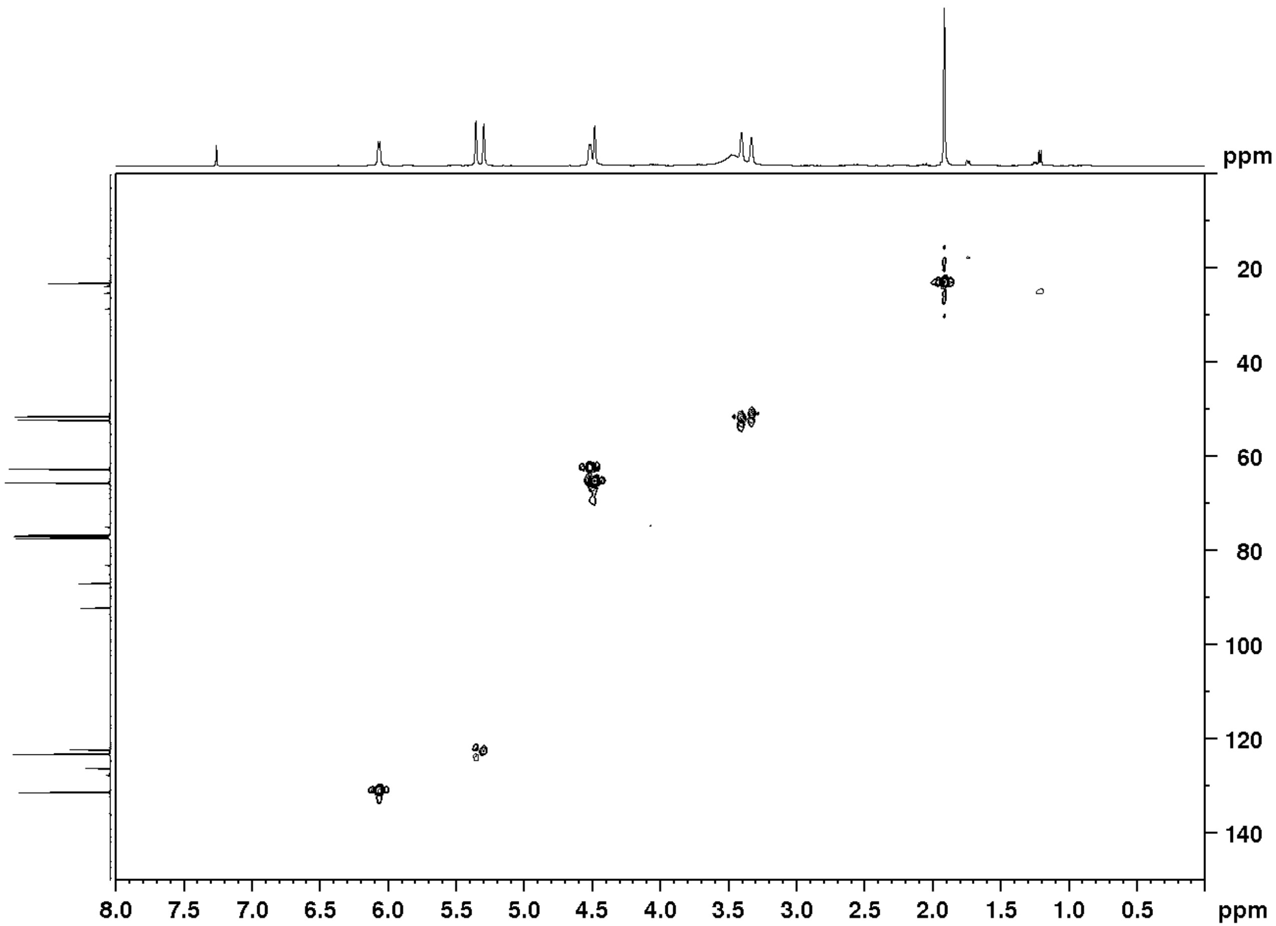

ed-HSQC spectrum of (–)-asperpentyn (**3**) (Bruker 400MHz, $CDCl_3$)

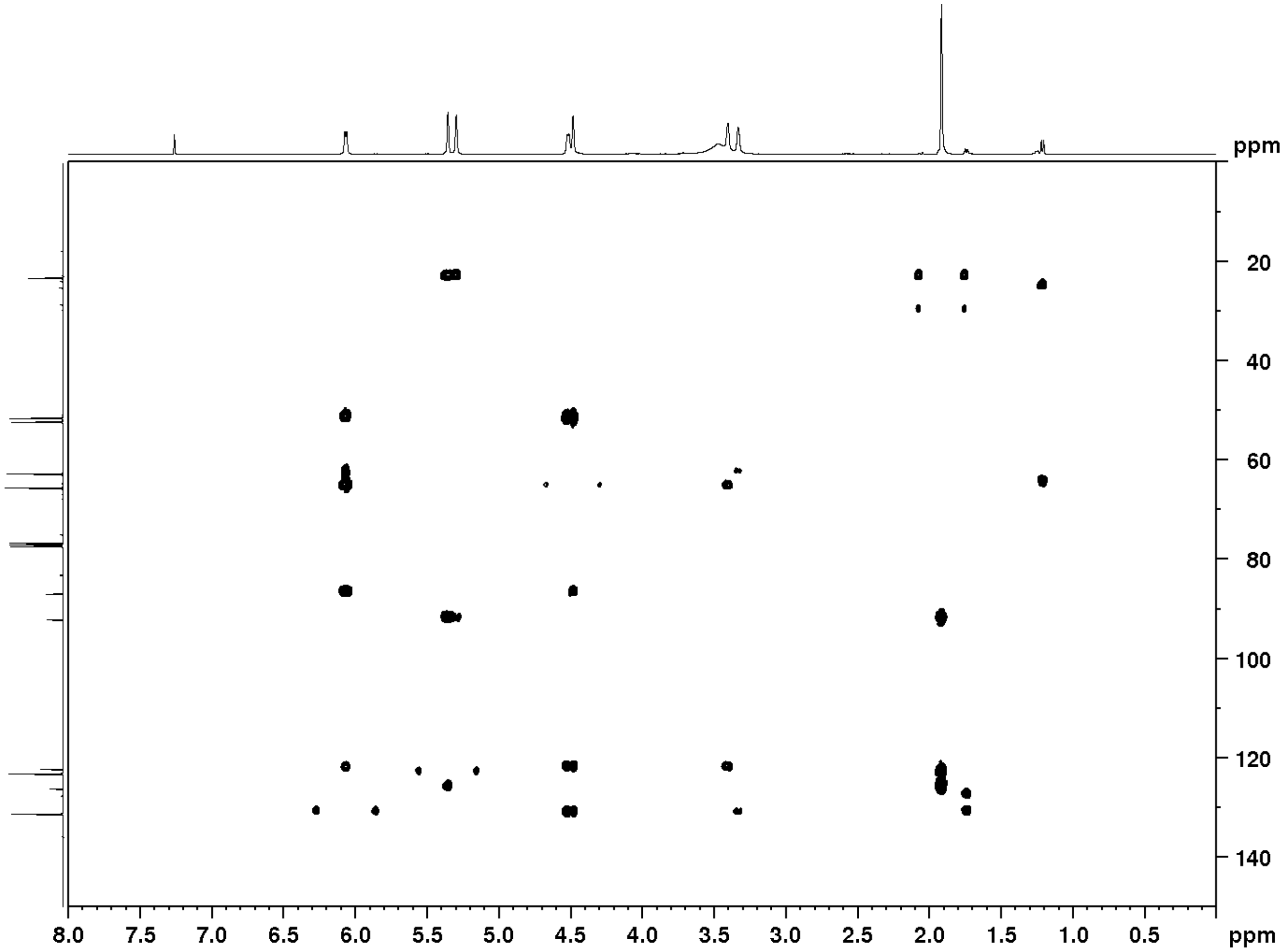

HMBC spectrum of (–)-asperpentyn (**3**) (Bruker 400MHz, $CDCl_3$)

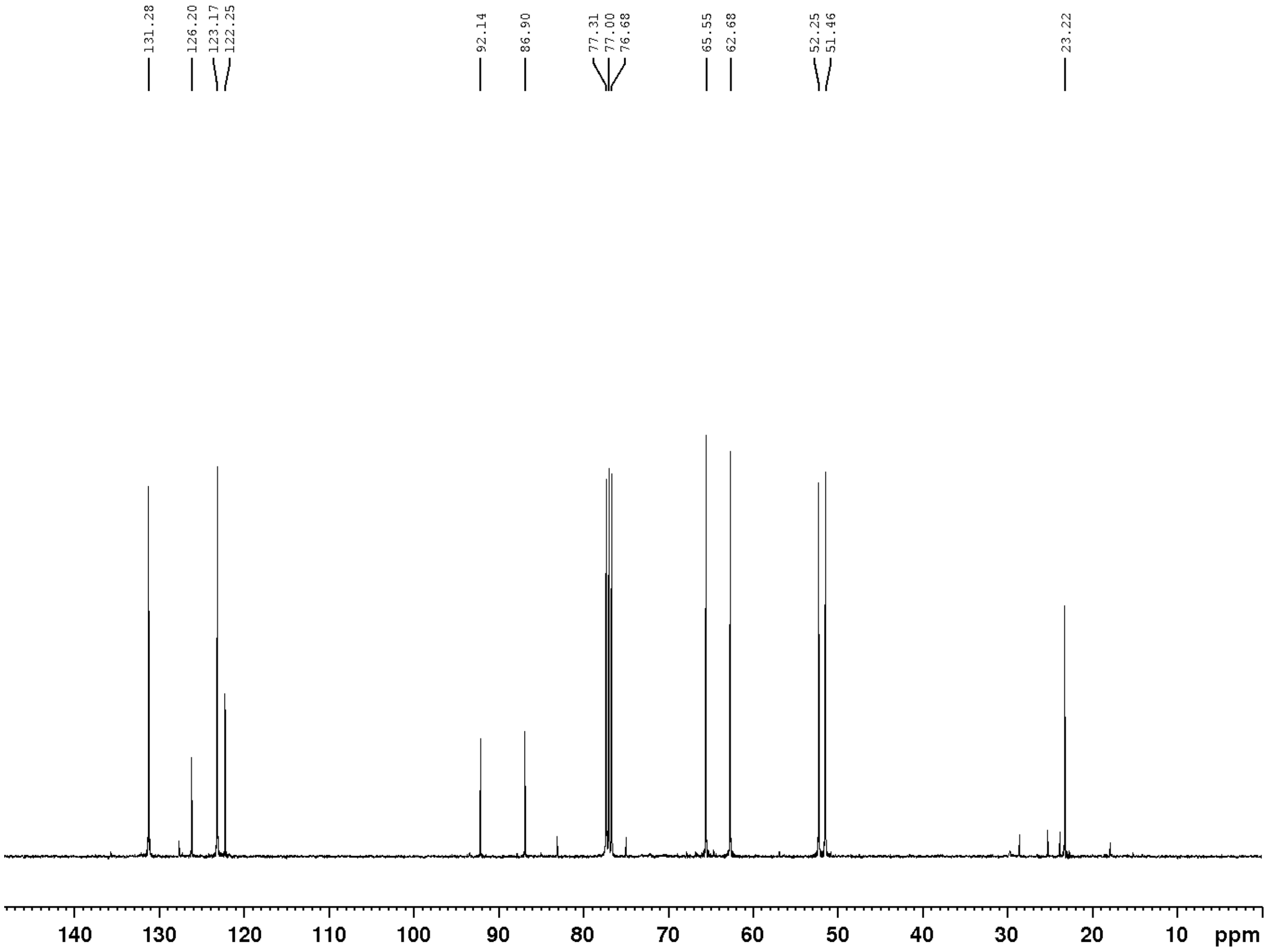

^{13}C NMR spectrum of (–)-asperpentyn (**3**) (Bruker 100 MHz, $CDCl_3$)

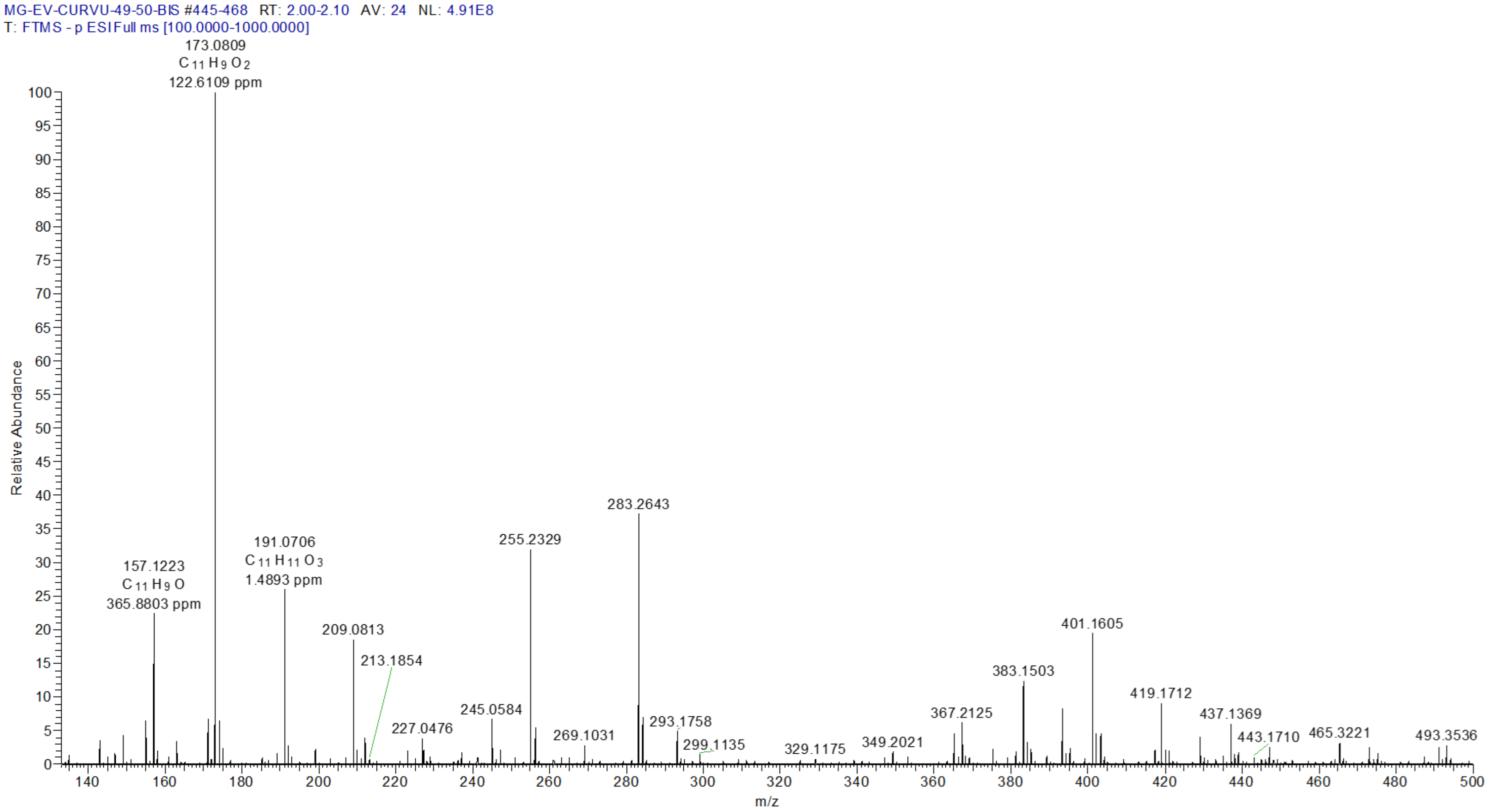

HR-(-)-ESIMS spectrum of (–)-asperpentyn (**3**)
